# Differential Impact of Substrate Peptides on Interdomain Interactions in Severe Acute Respiratory Syndrome Coronavirus 2 Main Protease

**DOI:** 10.34133/csbj.0058

**Published:** 2026-04-21

**Authors:** Asma Fatima, Kabir H. Biswas

**Affiliations:** College of Health and Life Sciences, Hamad Bin Khalifa University, Doha, Qatar.

## Abstract

Severe acute respiratory syndrome coronavirus 2 main protease (M^pro^) is essential for viral replication by cleaving polyproteins pp1a and pp1ab at 11 sites into functional proteins and remains a major pharmacological target. Although its structure and catalytic mechanism are well characterized, how different substrate peptides dynamically interact with and influence M^pro^ remains incompletely understood. To study these substrate-peptide-specific effects on M^pro^ structural dynamics, we used molecular dynamics (MD) simulations of the M^pro^ dimer bound to its cognate substrate peptides individually and complemented by analyses revealing substrate-peptide-specific structural changes in M^pro^. Specifically, we generated structural models of all M^pro^–substrate peptide complexes and performed all-atom, explicit solvent MD simulations. MD trajectory and SHapley Additive exPlanations (SHAP) analyses indicated that substrate peptides modulate M^pro^ dynamics in a substrate-specific manner, predominantly affecting the T45 to M49 and R188 to Q192 residues in the catalytic site. Importantly, this influence does not arise from a single conserved substrate position but from distinct residues across different substrate peptides, highlighting dynamic and context-dependent coupling. Furthermore, hydrogen bond (H-bond) interaction analysis showed substrate-peptide-specific differences in interdomain H-bond interaction between domains I and II. Together, these findings demonstrate that M^pro^ does not respond uniformly to substrate peptide binding; rather, each substrate peptide uniquely reshapes the flexibility of catalytic site residues and interdomain coupling, with potential implications for substrate recognition and inhibitor design.

## Graphical Abstract

**Figure:**
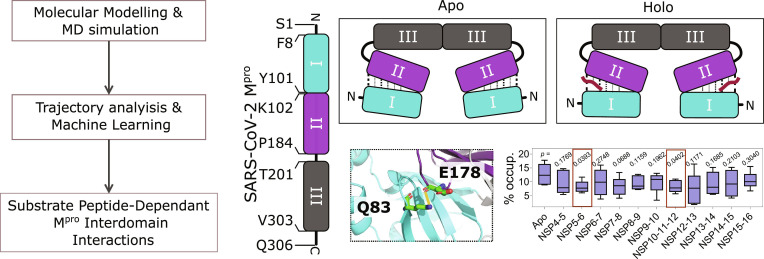

## Introduction

Nonstructural proteins (NSPs) of severe acute respiratory syndrome coronavirus 2 (SARS-CoV-2) are essential for viral replication and survival, as they are responsible for viral RNA synthesis, formation of the replication–transcription complex, modulation of host immune responses, and intracellular membrane remodeling [1–3]. Unlike structural proteins, NSPs are not synthesized as individual active units but are translated as 2 large inactive polyproteins, pp1a and pp1ab [4–6]. These polyproteins serve as inactive precursors that must undergo extensive and highly regulated proteolytic processing to generate 16 mature NSPs [6–8]. This proteolytic processing is carried out mainly by the papain-like protease and the main protease (M^pro^), also known as 3CL^pro^ [9–13]. Among the 2 SARS-CoV-2 proteases, M^pro^ plays a central role by cleaving the polyproteins at 11 conserved sites to release 12 functional NSPs [14,15]. Due to its essential role in NSP maturation and viral replication, M^pro^ is considered a key enzyme in SARS-CoV-2 infection cycle and an important target for antiviral drug development [14–16].

Structurally, M^pro^ functions as an obligate homodimer, with dimerization being essential for full catalytic activity [11]. Each protomer is composed of 3 distinct structural domains. Domains I (spanning residues F8 to Y101) and II (spanning residues K102 to P184) adopt a chymotrypsin-like fold, characterized by 2 antiparallel β-barrel structures, and together constitute the catalytic core of the enzyme [17,18]. The active site is located in the cleft between these 2 domains and harbors the highly conserved catalytic dyad H41 and C145, which mediates peptide bond hydrolysis during substrate processing [18,19]. The domain III (spanning residues T201 to V303) is predominantly α-helical, is connected to domain II by a flexible linker region (spanning residues F185 to I200), and plays a critical role in regulating dimerization by stabilizing interprotomer interactions [20–22]. Disruption of these interprotomer interactions substantially reduces M^pro^ catalytic activity, underscoring the allosteric coupling between domain III-mediated dimerization and enzymatic function [20,23,24]. Further, the substrate-binding pocket of M^pro^ is organized into discrete subsites, conventionally designated S4, S3, S2, S1, S1′, S2′, and S3′, which collectively govern substrate recognition and cleavage specificity [11,15,25]. Subsites S4 to S1 primarily accommodate residues on the N-terminal side of the scissile bond (P4 to P1) of the substrate peptide, whereas the S1′ and downstream subsites interact with residues on the C-terminal side (P1′ to P3′) [11,15].

Given its critical role in the SARS-CoV-2 infection cycle and the absence of a direct human homolog, M^pro^ has been identified as a particularly promising target for antiviral drug development [11,26–30]. M^pro^ inhibition effectively disrupts viral replication, while minimizing potential off-target effects on human cells, due to the absence of a close homolog in the human [31–33]. This focus has already borne fruit, exemplified by the rapid development and approval of Paxlovid, a combination therapy including nirmatrelvir, a potent and specific M^pro^ inhibitor, which has proven to be highly effective in treating COVID-19 [34–36].

**Fig. 1.**
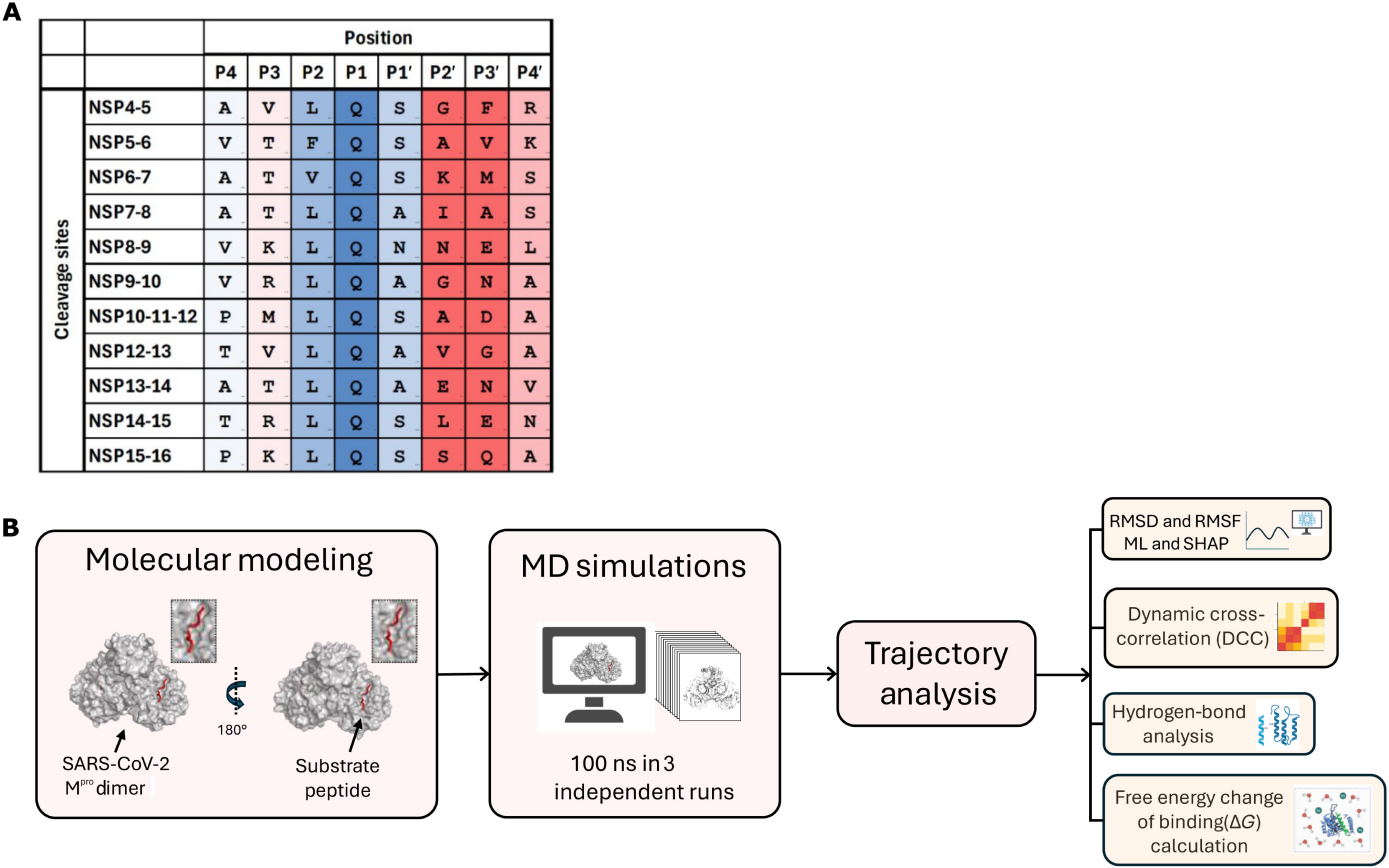
Main protease (M^pro^) substrate peptide sequence variation and M^pro^–substrate peptide complex modeling, molecular dynamics (MD) simulations, and computational analyses workflow. (A) Amino acid sequences of the 11 M^pro^ substrate peptides from the viral polyproteins, pp1a and pp1ab. Sequence variation is highlighted through background coloring from blue (lowest variation) to red (highest variation) showing maximum variation in P2′ and P3′ positions. (B) Workflow schematic showing M^pro^ dimer, either in the apo or in complex with individual substrate peptides bound to each of the catalytic sites, modeling, all-atom, explicit solvent MD simulation, and trajectory analysis. In total, 12 models were generated, including 1 apo dimer and 11 M^pro^–substrate peptide complexes. Each model was subjected to 3 independent, 100-ns-long MD simulations, and trajectories were analyzed for structural and dynamic features, including root mean square deviation (RMSD), root mean square fluctuation (RMSF), dynamic cross-correlation (DCC), free energy change of binding, and H-bond interactions. Extracted features were further interpreted through machine learning (ML) to identify key molecular determinants of M^pro^–substrate peptide interaction.

With regard to its functional activity, M^pro^ selectively cleaves viral polyproteins pp1a and pp1ab at conserved sites that are essential for viral maturation [8,37,38]. Its substrate preference follows a consensus cleavage motif, generally represented as (L/F/M)-Q↓(S/A/G/N), where the arrow denotes the scissile bond (Fig. 1) [37,39]. Within this motif, a glutamine (Q) residue at the P1 position, a hydrophobic residue such as L, F, or M at the P2 position, and a small aliphatic residue (S, A, G, or N) at the P1′ position are most critical for substrate peptide recognition and cleavage [37,39]. In contrast, the surrounding residue positions (P3, P4, and P3′) exhibit greater sequence variability, although they can modulate substrate binding affinity and stability [15]. Previous studies on SARS-CoV-2 M^pro^–substrate peptide interactions have primarily focused on extensively examining the interactions between substrate peptides and the M^pro^ catalytic site, using approaches such as x-ray crystallography, mutagenesis, enzymatic assays, and molecular dynamics (MD) simulations [11,37,38,40–42]. Due to the diverse range of amino acid variability at specific positions across the cleavage sites, several distinct types of interactions have been reported for different substrate peptides [15,37,42–46]. The binding of substrate peptides has the potential to alter the conformational dynamics of M^pro^ relative to its apo form [47]. However, it remains unclear whether different substrate peptides containing M^pro^ cleavage sites induce similar conformational changes in M^pro^ or whether substrate sequence variability differentially affects the dynamics of catalytic site residues and overall enzyme flexibility. To address this gap, we investigated and compared the structural dynamics of M^pro^ in complex with all its substrate peptides, asking whether the catalytic site residues exhibit substrate-specific dynamic adaptations.

We performed 3 independent, 100-ns-long, all-atom MD simulations of the M^pro^ dimer in complex with all its substrate peptides individually, followed by structural, energetic, and dynamic analysis. The analysis revealed that different substrate peptides induce distinct changes in the flexibility of the protease catalytic site, hydrogen-bond (H-bond) interactions and influence the stability of substrate peptide binding to the M^pro^ catalytic site. In addition, the flexibility of the substrate peptides themselves differentially influences the dynamics of the catalytic pocket. Two regions of M^pro^, encompassing residues T45, S46, M49, and R188 to Q192, exhibited differential sensitivity to substrate binding across the various M^pro^–substrate peptide complexes. Furthermore, substrate engagement altered the dynamic coupling of the catalytic site. Most importantly, H-bond interaction analysis showed the differences in interdomain interactions between domains I and II, with the catalytic pocket located at their interface. Notably, substrate-peptide-dependent variations were observed in interdomain H-bond interactions, including Q189–M49, N28–C145, N28 (side ND2)–C145, Y54–D187, Q19–N119, Q83–G178, and N84–G178. Together, these findings map how natural substrate sequence diversity drives distinct dynamic responses in M^pro^, providing a mechanistic framework for understanding substrate-specific modulation of protease structure and activity.

## Results and Discussion

### Structural modeling of M^pro^ in complex with substrate peptides

Given the differences in the amino acid residues between different M^pro^ substrate peptides (Fig. 1A), we aimed to study the changes in the structural dynamics of M^pro^ dimer in complex with different substrate peptides. For this, we generated structural models of the M^pro^ dimer in complex with its substrate peptides individually using Modeller [48] (Figs. S1 and S2). Modeller [48] was chosen over other protein modeling tools because it enables template-based modeling directly from experimentally determined structures, allowing precise placement of substrate peptides in the catalytic site. The substrate peptides were modeled as 8-mer sequences spanning the P4 to P4′ positions, a range selected to comprehensively map M^pro^ specificity while maintaining computational efficiency. While the full-length SARS-CoV-2 polyproteins pp1a (~4,400 amino acids) and pp1ab (~7,100 amino acids) represent the native biological context, their size renders them largely impractical for performing all-atom MD simulations [8]. In addition, structural studies confirm that M^pro^ derives its primary specificity from the core subsites S4, S2, S1, and S1′, all of which are fully occupied by the P4 to P4′ recognition envelope [15]. These 8-residue long sequence captures the evolutionarily constrained recognition envelope where molecular interactions are most densely localized near the scissile bond, particularly at the highly conserved P4 to P2′ positions [37]. Furthermore, previous high-resolution specificity profiling and high-throughput screening have validated the P4 to P4′ span as the experimental standard for M^pro^ functional studies, as residues further distal (e.g., P5 or P6) lack well-defined binding pockets and contribute minimally to substrate peptide binding and stabilization [49–51].

The quality of the generated models was evaluated using the discrete optimized protein energy (DOPE) score, a statistical potential that estimates the relative stability of protein structures, with lower scores indicating more thermodynamically favorable structural models (Table S1) [52]. Model quality was further assessed using Ramachandran plots via PROCHECK [53,54] (Figs. S1 and S2), and the secondary structure elements were visually inspected in PyMOL [55] to ensure proper folding and placement of the substrate peptides. We note that we also generated structural models for all 11 substrate-bound M^pro^ complexes using AlphaFold2, an artificial intelligence/deep learning-based protein structure prediction tool [56], and compared them with the structural models generated through the template-based approach using Modeller [48]. AlphaFold2 [56] predicted structural models exhibited reduced confidence in the substrate peptide regions, with mean predicted local distance difference test values of 46.40 ± 25.32, compared to 91.30 ± 5.58 for the M^pro^ dimer (Fig. S3 and Table S2). Moreover, structural alignment between Modeller [48]- and AlphaFold2 [56]-derived models revealed a backbone root mean square deviation (RMSD) of 3.3 ± 1.72 Å for the substrate peptide region, indicating notable conformational differences in the substrate binding pose (Fig. S4 and Table S3). These suggest an increased flexibility and lower predictive reliability for the substrate peptides in the AlphaFold2 [56] structural models. Based on these observations, Modeller [48]-derived structural models were used as the starting conformations for subsequent MD simulations to ensure the more reliable representation of substrate peptide binding within the catalytic pocket of M^pro^.

### Substrate-peptide-dependent modulation of M^pro^ catalytic site flexibility

**Fig. 2.**
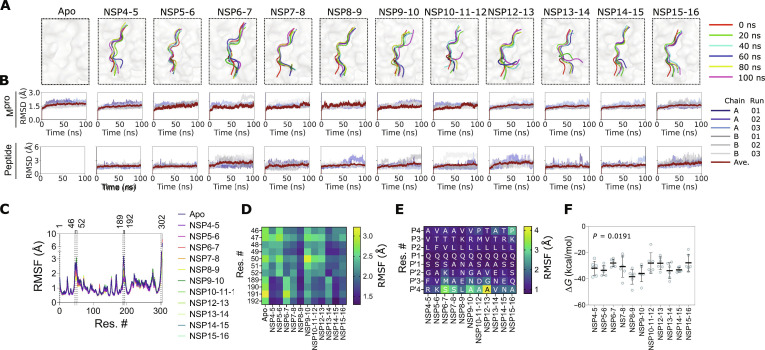
Differential structural dynamics of the main protease (M^pro^) in complex with its substrate peptide. (A) Snapshots of M^pro^ catalytic site (surface representation) in the absence and in the presence of indicated substrate peptide (cartoon representation) captured every 20 ns obtained from a representative 100-ns-long, all-atom, explicit solvent molecular dynamics (MD) simulation showing the orientation of the substrate peptides over the course of the simulation. (B) Graphs showing root mean square deviation (RMSD) values of individual monomers (top) and substrate peptides, if present, (bottom) from the apo or holo M^pro^ in complex with the indicated substrate peptide obtained from 3 independent 100-ns-long, all-atom, explicit solvent MD simulations. (C) Graphs showing average root mean square fluctuation (RMSF) values of M^pro^ residues in the apo state and in complex with indicated substrate peptides, from 3 independent 100-ns-long, all-atom, explicit solvent M^pro^ dimer MD simulations. Average values were calculated for each M^pro^ monomer and 3 independent simulation runs. (D and E) Heatmap showing average RMSF values of selected M^pro^ residues in apo and M^pro^–substrate peptide complexes (D) and substrate peptide residues (E) obtained from both monomers and 3 independent 100-ns-long, all-atom, explicit solvent M^pro^ dimer MD simulations. (F) Graph showing free energy change of binding (Δ*G*) of M^pro^ with the indicated substrate peptide. Data shown are means ± SD obtained from each monomer and 3 independent, 100-ns-long MD simulations, determined at 10-ns intervals. *P* value was obtained from a one-way ANOVA analysis.

Following structural modeling of the M^pro^ dimer with its substrate peptides, we then performed 3 independent, 100-ns-long, all-atom MD simulations individually for the apo and in complex with substrate peptides using the NAMD 2.13 software [57] and analyzed the MD simulation trajectories (Fig. 1B). In all the MD simulation trajectories, the substrate peptides were found to be bound to the M^pro^ catalytic site (Fig. 1B). We then assessed the RMSD of M^pro^ in both its apo form and in complex with substrate peptides (holo) to monitor structural stability over the simulation trajectories. In all simulation trajectories, the RMSD of M^pro^ dimer initially increased as the structure relaxed from the starting conformation and subsequently plateaued, indicating equilibration (Fig. 2B, top). Similarly, RMSD values of the substrate peptides bound to M^pro^ increased initially before stabilizing (Fig. 2B, bottom). The stabilization of the RMSD of both M^pro^ and substrate peptides confirms that the simulations were reliable and that the structures reached largely stable conformations suitable for further analyses of their structural dynamics (Fig. 2B).

Following the RMSD analysis, we determined the Cα atom root mean square fluctuation (RMSF) for both M^pro^ and the bound substrate peptides across the simulation trajectories to assess the impact of substrate peptide binding on the flexibility of the protease. RMSF provides a residue-level measure of atomic positional fluctuations relative to the average structure, thereby highlighting regions of increased mobility or rigidity. We compared the average RMSF values of M^pro^ in the apo and substrate-peptide-bound M^pro^ complexes (Fig. 2C and Fig. S5). This analysis revealed noticeable variations in the flexibility of the 2 regions of M^pro^ among M^pro^–substrate peptide complexes (Fig. 2D). The first region, spanning residues S46 to P52 and including S2 subsite residues T45, S46, and M49, showed high RMSF values in M^pro^ complexes containing NSP4-5, NSP5-6, NSP9-10, NSP10-11-12, and NSP12-13 substrate peptides. The S2 subsite forms a hydrophobic pocket that preferentially binds L at P2 in 9 of 11 substrate peptides, with smaller residues (e.g., V in NSP6-7 substrate peptide) fitting less optimally [15]. The NSP5-6 substrate peptide, which is also the C-terminal autocleavage site of M^pro^, has the residue F, containing a bulky side, at the P2 position [15]. In addition, it has been previously reported that residue T45 of S2 subsite shapes the catalytic pocket [58], the residue S46 contributes to H-bond interaction with P4 [15], and the residue M49 stabilizes the residue at the P2 position [59] of the substrate peptides. Together, these residues enable the catalytic site to accommodate the substrate peptides flexibly (Fig. 2D) [15,58,59]. The second region showing notable variation is the S6 to S3 region, which includes residues Q189 to Q192. In addition, the RMSF values of this region were higher in M^pro^ complexes with the NSP6-7, NSP7-8, NSP9-10, and NSP13-14 substrate peptides compared with those involving other substrate peptides (Fig. 2D). The residues Q189 to Q192 in M^pro^ form a flexible loop connecting M^pro^ domain II with domain III and are critical for substrate recognition and cleavage, with the residue Q189 making extensive van der Waals contact with and closely accommodating the residue at the P2 position in the substrate peptide [37]. In addition, the residue Q192 acts as an anchor and stabilizes the S4 pocket in M^pro^ [60]. The residues M190 and A191 are less directly involved in interaction with the substrate peptide [37]. However, mutations at these positions have been reported to alter protease cleavage kinetics, likely by affecting loop positioning and the orientation of neighboring residues such as residues Q189 and Q192 [61].

Following analysis of the M^pro^ residue fluctuations, we assessed the fluctuations of M^pro^ substrate peptide residues within the catalytic pocket by calculating the average RMSF values for each residue in all the substrate peptides and compared the values at equivalent sequence positions (Fig. 2E). Notably, the residue at P1 position (the conserved Q residue in all substrate peptides) exhibited markedly lower RMSF values compared to the other positions within the substrate peptides (Fig. 2E). This reduced flexibility likely reflects its critical role in substrate recognition and stable positioning at the catalytic site of M^pro^ for proteolytic cleavage. In contrast to the P1 position, residues at both the N and C termini of the substrate peptides exhibited higher RMSF values compared to residues near the cleavage site (P1 to P1′) (Fig. 2E). A direct comparison revealed that the C-terminal residues (P2′ to P4′ positions) were generally more flexible than the N-terminal residues (P4 to P2 positions) (Fig. 2E). Among the C-terminal positions, NSP6-7 substrate peptide displayed the highest overall flexibility, followed by those in NSP7-8, NSP9-10, NSP10-11-12, and NSP12-13 substrate peptides. Within this group, the C-terminal P4 residue of NSP12-13 substrate peptide showed the highest value of RMSF, representing the highest fluctuation among residues at this position (Fig. 2E). While the N-terminal residues showed relatively less flexibility across different substrate peptides, the residue at the P4 position (residue P) in substrate peptide NSP15-16 substrate peptide exhibited higher RMSF relative to other N-terminal positions.

To further evaluate the stability of substrate peptide binding to the M^pro^ dimer, we determined the free energy change of binding (Δ*G*) of the M^pro^–substrate peptide complexes using the molecular mechanics Poisson–Boltzmann surface area method [62]. The computed Δ*G* values reflect the overall thermodynamic favorability of substrate binding, with more negative values indicating more stable binding within the M^pro^ catalytic site. There were significant differences in the Δ*G* values among the M^pro^–substrate peptide complexes (one-way analysis of variance [ANOVA], *P* = 0.0191) (Fig. 2F). The average Δ*G* was most favorable for the NSP8-9 substrate peptide, indicating stronger and more stable interactions of this substrate peptide compared to the other substrate peptides (Fig. 2F). Notably, M^pro^ in complex with NSP8-9 substrate peptide also exhibited reduced flexibility in key catalytic site regions (residues S46, M49, Y54, and Q189 to Q192) and in the substrate peptide residues themselves, compared to other M^pro^–substrate peptide complexes (Fig. 2D and E). These observations suggest that stronger substrate peptide binding is associated with localized stabilization of catalytic site regions, providing a link between binding energetics and substrate-induced modulation of local dynamics.

### Substrate peptide residues selectively modulate the flexibility of key M^pro^ catalytic site regions

**Fig. 3.**
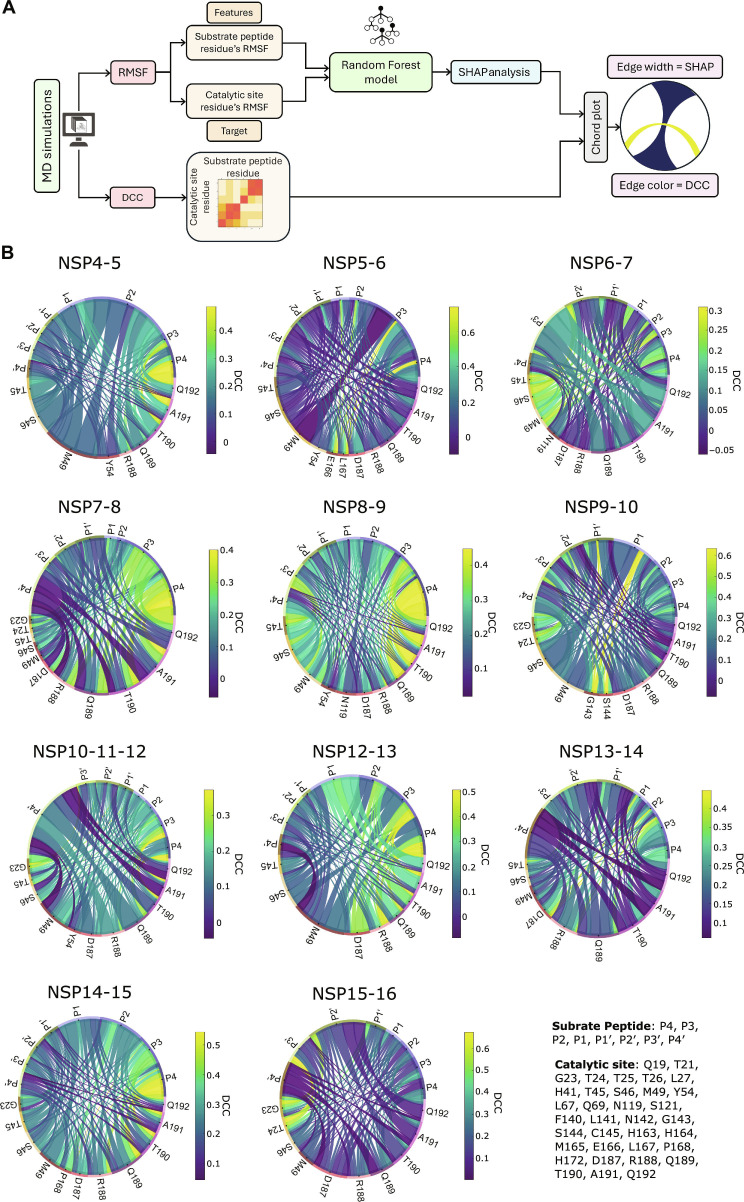
Substrate peptide residues govern the flexibility of main protease (M^pro^) catalytic site at distinct regions. (A) Schematic illustrating the workflow for SHapley Additive exPlanations (SHAP) analysis and generation of chord plots using SHAP analysis data and dynamic cross-correlation (DCC) matrix. SHAP analysis was performed using root mean square fluctuation (RMSF) data obtained from each monomer and 3 independent 100-ns-long molecular dynamics (MD) simulations, while the DCC matrices used were the average of each monomer and 3 independent 100-ns-long MD simulations. (B) Chord plots of the SHAP value matrix from SHAP analysis and the average DCC matrix of the indicated M^pro^–substrate peptide complex, highlighting only catalytic site residues with an overall mean SHAP value greater than 0.15. Note that the plots illustrate how the flexibility of substrate peptides influences the M^pro^ catalytic site and depict the dynamic correlations between catalytic site and substrate peptide residues across M^pro^–substrate peptide complexes.

The flexibility of substrate peptides can influence the dynamics of catalytic site residues and vice versa [63,64]. Our RMSF analysis revealed distinct differences in the flexibility of the 11 substrate peptides bound to the M^pro^ catalytic site. These differences likely arise from variations in amino acid sequence and the resulting interactions with catalytic site residues, suggesting that substrate fluctuations may contribute to changes in catalytic residue dynamics. To investigate this, we applied machine learning to explore how substrate residue flexibility impacts catalytic site residue flexibility. Importantly, the goal was to uncover patterns in the data rather than to make predictions. We trained Random Forest Regression models using substrate peptide residue RMSF as input features and catalytic site RMSF as target variables. Random Forest is a supervised learning algorithm that constructs an ensemble of decision trees [65], making it well suited to capture the complex, nonlinear relationships between the fluctuations of individual substrate residues and the resulting changes in catalytic site residues, relationships that simpler linear models or traditional statistical analyses may fail to capture. To interpret the trained models, we used SHAP (SHapley Additive exPlanations), which quantifies each input feature’s contribution to the model’s output [66]. Separate models were trained for each catalytic residue, and SHAP values were calculated for each M^pro^–substrate peptide complex. The performance of trained models was evaluated using *R*^2^, where higher values indicate stronger predictive performance. The models showed robust overall performance (*R*^2^ = 0.70 to 0.98), with a single outlier observed for the NSP12-13 substrate peptide with catalytic site residue N119 (*R*^2^ = 0.62) (Table S4 and Fig. S6). We next generated chord plots to visualize the SHAP analysis results combined with dynamic cross-correlation (DCC), which was performed using the MD-TASK suite to evaluate correlated motions between residues over time (Fig. 3A). The analysis revealed the correlation in motion between the substrate peptide residues and the catalytic site residues, where positive values indicate coordinated motions, negative values indicate opposing motions, and values near zero indicate largely independent fluctuations (Fig. 3A and Fig. S7). To aid visualization, we applied a SHAP cutoff (0.15) to include only catalytic site residues most affected by substrate peptide flexibility. In the chord plots, ribbon thickness represents SHAP importance, while ribbon color indicates DCC between substrate peptide and catalytic site residues (Fig. 3A).

Our SHAP analysis indicateed that the substrate peptide flexibility modulates the M^pro^ catalytic site in a nonuniform and substrate-peptide-specific manner (Fig. 3B). SHAP-based interpretation of the machine learning models identified 2 primary sensitivity regions of the catalytic site: the S3/S4 subsite spanning residues R188 to Q192, which is consistently perturbed by most substrates, and a secondary region comprising T45, S46, and M49 that showed more selective responsiveness. Substrate peptides such as NSP6-7, NSP8-9, NSP14-15, and NSP15-16 influenced both regions simultaneously, suggesting a coordinated modulation of the active-site pocket. In contrast, NSP7-8 and NSP13-14 substrate peptides preferentially targeted the R188–Q192 region with minimal impact on the T45/S46/M49 cluster, whereas NSP4-5, NSP5-6, NSP9-10, and NSP12-13 substrate peptides primarily perturbed the residues T45/S46/M49. Beyond these dominant trends, NSP6-7 substrate peptide selectively modulated the residue N119, NSP9-10 substrate peptide perturbed the G143 and S144, and NSP15-16 substrate peptide prominently influenced the residue D187 (Fig. 3B). We note that previous cocrystal structures of M^pro^ H41A mutant with 6 cognate substrate peptides showed that R188–A191 loop shifts outward, while residue pairs R188 and Q189 and Q189 and Q192 stabilized the S2/S4 subsites through hydrophobic contacts directed by the residue at P2 position and main-chain H-bond interactions [15].

We also observed the drivers of these effects vary between substrate peptides, as the NSP4-5, NSP5-6, NSP6-7, and NSP10-11-12 substrate peptides are dominated by the flexibility of a single substrate position, while NSP12-13 and NSP8-9 substrate peptides exhibit a more distributed influence involving multiple substrate positions (Fig. 3B). Previous reports have shown that the L residue at P2 position can reshape the S2 subsite by shifting the orientations of M49 and Q189, whereas, in our analysis, only NSP4-5 displayed a strong P2-driven effect on the flexibility of these residues. However, 9 of 11 substrate peptides have an L residue at the P2 position. This difference may have come from the use of static crystal structures of the M^pro^ C145A or H41A mutant bound to substrate peptides, structures that may carry mutation-induced allosteric effects, while our approach captures the dynamic behavior of the wild-type M^pro^ [15,51].

Beyond these dominant trends, the NSP6-7 substrate peptide selectively modulated the residue N119, NSP9-10 substrate peptide perturbed the oxyanion-hole forming residues G143 and S144, and NSP15-16 substrate peptide prominently influenced the residue D187. Consistent with these observations, DCC analysis revealed generally weak correlation between substrate peptide and catalytic site residues. However, stronger, substrate-specific correlations were observed for the NSP5-6 and NSP15-16 substrate peptides, as well as between prime-side residues and the T45/S46 cluster in NSP8-9 and NSP14-15 complexes (Fig. 3B). Overall, DCC analysis suggests that catalytic site residues with high SHAP importance show generally weak positive correlations with substrate residues, with notable exceptions for specific pairs. In the NSP5-6 substrate peptide complex, P3 and P3′ residues correlated strongly with E166 and L167, while, in the NSP15-16 substrate peptide complex, P3′ showed strong correlation with T24 motion (Fig. 3B). In conclusion, these analyses suggest that substrate peptides selectively modulate specific regions of the M^pro^ catalytic site, with effects varying by residue and subsite.

### H-bond analysis between the M^pro^ catalytic pocket and substrate peptides

**Fig. 4.**
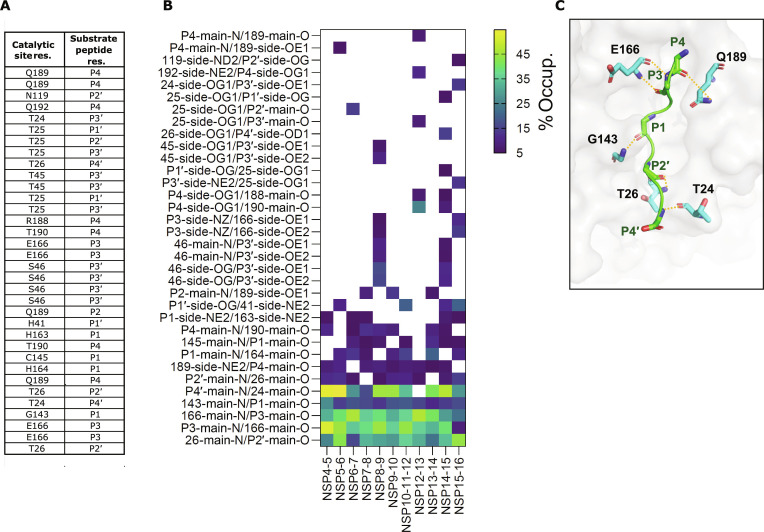
Substrate peptide specific variations in H-bond interactions between main protease (M^pro^) and substrate peptides. (A) Table listing the M^pro^ and substrate peptide residues involved in H-bond interactions between M^pro^ and substrate peptides. (B) Heatmap showing the percentage occupancy of H-bond interactions between M^pro^ and substrate peptide residues. The percentage occupancy shown is the average of the 2 monomers and 3 independent, 100-ns-long molecular dynamics (MD) simulations. (C) Surface representation of the M^pro^ catalytic pocket and the substrate peptide in a cartoon representation showing H-bond interactions common across multiple substrate peptides (more than 10; highlighted in orange dashed lines between donor and acceptor residues, shown as sticks).

To further investigate the differences in the interaction between the distinct substrate peptides with M^pro^ catalytic sites, we analyzed the M^pro^–substrate peptide complex MD simulation trajectories for H-bond formation between M^pro^ and the substrate peptide residues. This analysis revealed a total of 34 distinct H-bond interactions between the M^pro^ and substrate peptide residues after the application of a minimum occupancy threshold of 5% (Fig. 4A). Across all the M^pro^–substrate peptide complexes, we identified 5 H-bond interactions formed in a minimum of 10 substrate peptides: P4′–T24, G143–P1, E166–P3, P3–E166, and T26–P2′ (donor–acceptor), which involved main-chain atoms, indicating their role in maintaining stable binding of the peptides to M^pro^ catalytic site (Fig. 4B). For example, the backbone N–H of residues G143, E166, and T26 formed H-bond interactions with the substrate peptide carbonyl oxygen of residues at P1, P3, and P2′ positions, respectively. The main-chain carbonyl of residues E166 and T24 formed H-bond with the amide group at the P2′ position of the substrate peptides, across all the 11 M^pro^–substrate peptide complexes (Fig. 4C). In addition, the backbone carbonyl of residue T24 formed an H-bond with the residue at P4′ position in the substrate peptides in all M^pro^–substrate peptide complexes except for the NSP12-13 substrate peptide (Fig. 4B).

In contrast to these conserved interactions, several catalytic site residues exhibit high substrate specificity, forming H-bond interactions with only a few substrate peptides (Fig. 4B). The residue N119 in the S1′ subsite exhibited H-bond interaction with the S residue at P2′ position in the NSP15-16 substrate peptide complex. In the S4 subsite, Q192 and R188 interact with the side chain of the T residue at P4 position exclusively in the NSP12-13 and NSP14-15 substrate peptide complexes. Shaqra et al. [37] also reported H-bond interaction involving R188 between NSP12-13 substrate peptide and M^pro^ C145A mutant. In addition, the side chain of one of the catalytic dyad residues, H41, was observed to be an H-bond acceptor for the S residue at P1′ position in the NSP5-6, NSP10-11-12, NSP14-15, and NSP15-16 substrate peptides (Fig. 4B). Although NSP4-5 and NSP6-7 substrate peptides also possess an S residue at P1′ position, no H-bond interaction with H41 was detected with these substrate peptides in our analysis. It is possible that transient or low-occupancy H-bond interactions occur but are not captured because of the stringent cutoff applied in the analysis. The residue H41 has been reported to participate in π–π stacking interactions with substrate peptides [46]. Notably, catalytic site residues with high SHAP values (T45, S46, M49, Y54, and D187 to Q192) frequently participated in these specific H-bond interactions. For instance, the Q189 side chain formed H-bond interactions with the residue at P4 position carbonyl oxygen in 9 of 11 complexes, while the N–H of T190 interacted with residue at P4 position in 8 complexes (Fig. 4B). In addition, T45 and S46 formed several H-bond interactions with residues at P3′ and P4′ positions only in the NSP8-9 and NSP14-15 substrate peptides (Fig. 4B). For the NSP8-9 substrate peptide, the reduced fluctuations and more favorable Δ*G* observed above are consistent with the higher number of H-bond interactions seen here, supporting a stronger binding and a localized stabilization of the catalytic region. While some of the observed H-bond interactions were detected in these high SHAP regions, others, such as M49 and D187, showed no stable direct contact. This suggests that the substrate-peptide-driven effects captured by SHAP analysis likely arise from a combination of specific H-bond interaction and the indirect modulation of local or long-range interaction networks within the M^pro^ catalytic site.

### Substrate binding reshapes long-range correlated motions in M^pro^

While H-bond interaction analysis confirmed consistent interaction patterns between the various substrate peptides and the catalytic site residues, our RMSF and SHAP analysis identified 2 specific protease regions exhibiting differential fluctuations depending on the bound substrate peptide. To investigate whether these fluctuations arise from long-range allosteric modulation rather than direct steric contacts, we performed DCC analysis. This method quantifies the degree of correlated motion between residue pairs over time, where a coefficient of 1.0 indicates perfectly correlated movement.

**Fig. 5.**
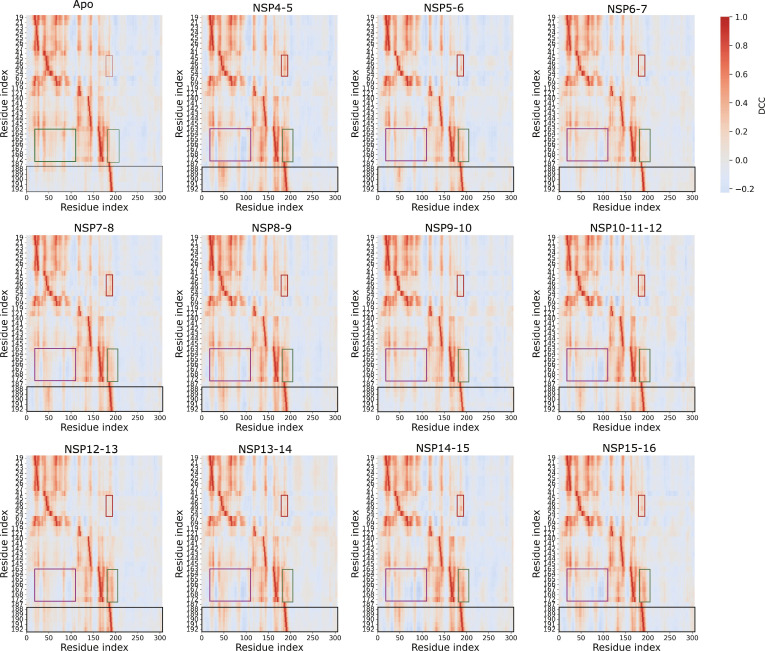
Dynamic cross-correlation (DCC) analysis of main protease (M^pro^) catalytic site residues. Heatmaps illustrating the DCC of Cα fluctuations between catalytic site residues (*y* axis) and the full-length M^pro^ (*x* axis) for the apo form and substrate-bound complexes. Regions exhibiting notable differences in correlation patterns between apo M^pro^ and substrate-bound states are highlighted by colored boxes.

A comparison of the apo M^pro^ and the substrate-peptide-bound complexes revealed that substrate peptide occupancy fundamentally reshapes the internal correlated motions within the protease. We observed that several residues in the catalytic subsite change their correlation patterns with other M^pro^ residues upon binding. A set of catalytic site residues (M165 to P168 and H172), which are located near to the residues R188 to Q192 (distal loop), are more positively correlated with domain I (highlighted with a purple box) and less correlated with the distal loop and its surrounding residues in the apo state (highlighted with a green box) (Fig. 5). However, in the holo M^pro^ dimer, these residues showed a decrease in positive correlation with domain I, and instead, the intensity of negatively correlated motions with this domain increased (Fig. 5). In addition, residues M165 to P168 and H172 become more positively correlated with the distal loop and neighboring residues in the bound states. This shift is not observed for the NSP6-7 and NSP9-10 substrate peptide complexes (Fig. 5). Furthermore, several S2 subsite residues (T45, S46, M49, and Y54) showed an increase in positively correlated motion with the residues R188 to Q192 and their surrounding residues upon substrate binding (highlighted with a red box) (Fig. 5). Interestingly, the local correlation profile of residues R188 to Q192 transitions from very low correlated motion with domain I and domain II in the apo M^pro^ to a highly correlated profile across all substrate-bound states (highlighted with a black box) (Fig. 5). These results connect directly to the functional importance of the R188–Q192 loop, which plays a key role in substrate recognition, and inhibitor binding, largely through H-bond interactions and local loop flexibility [67,68]. Structural and mutational studies show that substitutions such as Q192V disrupt H-bond interaction networks with V186 and R188, diminishing the efficacy of inhibitors such as nirmatrelvir, while R188 engages directly with the L residue at P2 position of peptidomimetic substrates [67]. The MD simulations further reveal that antiviral compounds, including ribavirin, form H-bond interactions with R188 and T190, underscoring their plasticity and potential for allosteric modulation [69]. Moreover, resistance-associated variants frequently harbor mutations near residues R188 to Q192, highlighting this region as a critical hotspot for inhibitor design [70,71].

### Distinct substrate peptides differentially impact the interdomain H-bond interactions of M^pro^

**Fig. 6.**
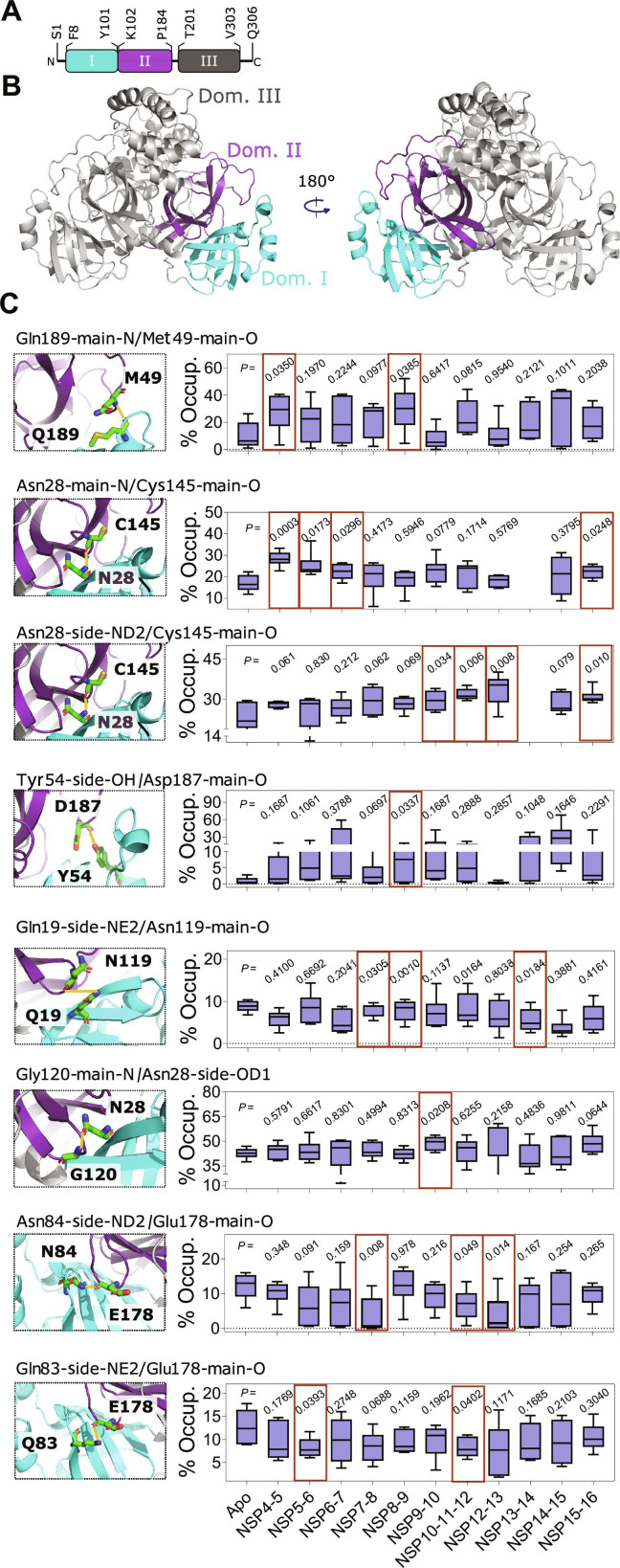
Substrate-peptide-dependent modulation of interdomain H-bond interactions in main protease (M^pro^) catalytic site. (A) Schematic of M^pro^ showing its 3 domains: domain I (residues 8 to 101), domain II (residues 102 to 184), and domain III (residues 201 to 303). (B) Cartoon representation of an M^pro^ dimer showing domain II in cyan and domain III in purple. The left structure shows the catalytic site viewed from the M^pro^–substrate peptide interface (front), while the right panel shows the same site rotated 180°. (C) Left: Representative snapshots of M^pro^ showing specific residue pairs involved in H-bond interaction (green stick representation), with H-bond interactions highlighted with orange lines. Right: Box-and-whisker plots showing % H-bond occupancy for each specific residue pair in the indicated M^pro^–substrate peptide complex. The box represents the interquartile range (25th to 75th percentile), the horizontal line within the box indicates the median, and the whiskers extend to the 5th and 95th percentiles. Percentage occupancy values were calculated for each monomer and 3 independent, 100-ns-long molecular dynamics (MD) simulations of the apo M^pro^ and M^pro^–substrate peptide complex dimers. Maroon boxes on the plots indicate M^pro^ with substrate peptide complexes that showed significant differences in % H-bond occupancy relative to apo M^pro^*. P* values were obtained from Student’s *t* test, comparing the % H-bond occupancy of the apo M^pro^ and the indicated M^pro^–substrate peptide complex.

To further investigate the impact of substrate peptides on the local H-bond interactions between the catalytic site residues, we analyzed the MD simulations trajectories of the apo and substrate-peptide-bound M^pro^ for H-bond interactions between domain I and II of M^pro^ (Fig. 6A, B). This analysis aims to reveal any changes in occupancy of H-bond interactions that may arise from substrate binding. We included only those interactions that were observed consistently in most complexes during the MD simulations. Interdomain H-bond interactions observed in the M^pro^–substrate peptide complexes were compared with those in the apo M^pro^. This analysis resulted in the identification of 8 interdomain H-bond interactions that were present in all the M^pro^ structures, whether in apo form or bound with substrate peptides (Fig. 6C). These included H-bond interactions such as Q189–M49, N28–C145, N28 (side ND2)–C145, Y54–D187, Q19–N119, Q83–G178, and N84–G178. Importantly, several M^pro^–substrate peptide complexes showed significant differences in occupancy of these H-bond interactions compared to their occupancy in apo M^pro^ (Fig. 6C). For instance, the H-bond interaction between residues Q189 and M49 displayed a significantly higher occupancy in M^pro^ bound with NSP4-5 (*P* = 0.035) and NSP8-9 (*P* = 0.039) substrate peptides, while H-bond interaction between residues Y54 and D187 was only found to be significantly higher in M^pro^ with NSP8-9 substrate peptide (*P* = 0.034) (Fig. 6C and Table S5). Interestingly, 2 of the interdomain H-bond interactions involved residue C145, a key residue of the M^pro^ catalytic dyad essential for the proteolytic activity of M^pro^ [72]. Specifically, the main-chain oxygen of residue C145 formed 2 interactions: one with the main-chain nitrogen of residue N28 and another with the ND2 atom of residue N28. However, these H-bond interactions were not consistently observed across all complexes. Notably, M^pro^ with NSP13-14 substrate peptide complex did not show these H-bond interactions. In the remaining M^pro^–substrate peptide complexes, namely, NSP4-5, NSP5-6, NSP6-7, NSP9-10, NSP10-11-12, and NSP15-16 substrate peptides, the occupancy of these interactions was significantly higher than in the apo M^pro^ (*P* < 0.001 and *P* = 0.017, 0.030, 0.034, 0.006, 0.008, and 0.010, respectively) (Fig. 6C and Table S5). In addition, N28 formed a H-bond interaction with residue G120 of domain II, with occupancy differing notably only in the M^pro^–NSP9-10 substrate peptide complex relative to the apo M^pro^ (*P* = 0.034) (Fig. 6C and Table S5). In addition to these interactions, another H-bond interaction that is formed between residues Q19 and N119 showed significantly higher occupancy in M^pro^–NSP7-8 (*P* = 0.031), M^pro^–NSP8-9 (*P* = 0.001), and M^pro^–NSP10-11-12 (*P* = 0.016) M^pro^–substrate peptide complexes, as compared to the apo M^pro^ (Fig. 6C and Table S5).

Furthermore, 2 additional H-bond interactions were observed between residue pairs Q83–G178 and N84–G178 that are located adjacent to the catalytic site. Interestingly, the occupancy of these H-bond interactions was lower in the M^pro^-substrate peptide complexes than in the apo M^pro^. Among the M^pro^–substrate peptide complexes, the Q84–G178 H-bond showed significantly less occupancy in the M^pro^–NSP7-8 (*P* = 0.008), M^pro^–NSP10-11-12 (*P* = 0.049), and M^pro^–NSP12-13 (*P* = 0.014) complexes. On the other hand, the N83–G178 H-bond exhibited differences in occupancy in the M^pro^–NSP5-6 (*P* = 0.039) and M^pro^–NSP10-11-12 (*P* = 0.04) complexes (Fig. 6C and Table S5). The residues Q83, N84, and G178 are positioned behind the catalytic pocket and are not part of the catalytic site. Neither the literature nor our analyses indicate any direct interaction between these residues and the substrate peptides [67,68]. In the apo state, the Q83/N84–G178 H-bond interaction showed high occupancy because this interdomain region remains relatively stable and unobstructed. In the presence of the substrate peptide, however, the structural rearrangements required to accommodate the peptide within the catalytic pocket propagate toward the domain I and domain II interface. These subtle but consequential shifts may reduce the stability of the Q83/N84–G178 hydrogen bond.

To assess whether substrate peptide binding induces global conformational shifts, we measured the center-of-mass (COM) distance between domains I and II. The COM distance distributions of M^pro^–substrate peptide complexes were similar to the apo state. Mean COM values remained within a narrow range (~12.2 to 12.9 Å), and most differences were in the range of ~0.1 to 0.2 Å, indicating that interdomain spacing is largely conserved with only subtle changes across M^pro^ substrate peptides (Fig. S8). Furthermore, DCC analysis showed that overall interdomain communication between domains I and II remains largely conserved; however, substrate peptide binding induceed localized, substrate-specific perturbations, predominantly centered around residues Y161 to V171 (Figs. S9 and S10). Importantly, the magnitude of dynamic reorganization varied across complexes, revealing a gradient of allosteric sensitivity, where certain complexes (e.g., with NSP15-16 and NSP12-13 substrate peptides) exhibited stronger coupling changes, while others remained similar to apo M^pro^ (e.g., NSP6-7, NSP8-9, and NSP10-11-12 substrate peptides), suggesting selective modulation rather than global structural rearrangement (Figs. S9 and S10). At the residue level, substrate binding induced significant changes in the DCC of C145 with both catalytic site and distal residues. Notably, complexes exhibiting increased H-bond occupancy between residues C145 and N28 showed a greater number of residues with altered correlation to C145. This may also suggest that substrate-specific interdomain H-bond rearrangements are coupled to coordinated changes in both domain-level motions and catalytic site dynamics, supporting a mechanistic link in which modulation of interdomain interactions propagates through the protein to reshape the dynamic coupling network underlying catalysis (Fig. S11).

### Limitations of the study

MD simulations of the M^pro^ dimer with individual, cognate substrate peptides performed in the current study elucidated differences in the structural dynamics of the protease. However, we note that we have used short, 8-residue-long substrate peptides rather than the full-length SARS-CoV-2 polyproteins. While these peptides effectively captured the core recognition motifs required for M^pro^ substrate binding and enabled systematic, computationally tractable simulations, they cannot account for potential long-range or allosteric interactions present in the native polyprotein substrates. The full polyproteins pp1a (~4,400 amino acids) and pp1ab (~7,100 amino acids) are too large for all-atom MD simulations and lack experimentally resolved structures in complex with M^pro^; consequently, distal regions that may influence M^pro^ conformation or cleavage dynamics are not captured in the peptide-based models used here. In addition, while the 100-ns-long trajectories used in this study are sufficient to capture local atomic fluctuations and H-bond interaction dynamics, they may not fully sample slower conformational transitions, and longer simulations are required to assess larger substrate-induced conformational changes. Finally, the machine learning analysis is limited by the small dataset and reliance on time-averaged RMSF values, restricting its use to feature interpretation rather than direct prediction of dynamic fluctuations.

## Materials and Methods

### Molecular modeling

A total of 11 M^pro^ dimer 3-dimensional (3D) models were generated, each representing a distinct substrate peptide bound to the catalytic site. Each dimer consisted of 2 identical monomers, and the only difference among the models was the substrate peptide bound to the catalytic site of each monomer. The 3D structures were generated using a homology modeling tool, Modeller (version 10.4) [48]. For the template structure file, the M^pro^ C145A mutant dimer in complex with the NSP9-10 substrate peptide structure (Protein Data Bank ID: 7TA4) [37] was downloaded from RCSB database (https://www.rcsb.org). For each complex, individual ALI (alignment) files were prepared by substituting A145 in the M^pro^ dimer sequence with C145 and the M^pro^–NSP9-10 substrate peptide with the respective substrate peptide sequence. Qualities of the structural models were assessed using the DOPE score, a statistical potential parameter that estimates the relative stability of protein structures [52], with lower DOPE scores indicating more thermodynamically favorable and reliable models (Table S1) [52]. The quality of the models was further assessed by inspection of the Psi/Phi Ramachandran plots obtained using PROCHECK analysis (https://saves.mbi.ucla.edu) [53,54] (Figs. S1 and S2). Last, the secondary structure elements in the structural models were inspected manually using PyMOL [55]. In addition, we also predicted the 3D structures of M^pro^–substrate peptide using artificial-intelligence-based modeling approach with AlphaFold2 [56]. However, the substrate peptides in models had lower confidence in the substrate peptides, as indicated by lower predicted local distance difference test scores and greater conformational heterogeneity. RMSD values between Modeller [48] and AlphaFold2 [56] M^pro^–substrate peptide structural models were calculated using PyMOL [55].

### MD simulation and analysis

MD simulations of either the apo or M^pro^ dimer in complex with different substrate peptides were performed using the NAMD 2.13 software [57] and the CHARMM36 force field [73]. CHARMM-GUI online server [74] was used to generate the input topology and parameter files. First, the TIP3P cubic water box [75] was used to dissolve the protein in an explicit solvent environment. The minimum distance between the edges of the box and any of the atoms of the protein complex was set to 10 Å. Further, 0.15 M NaCl was introduced into the solvated system. Before initiating the production run, the simulation system was subjected to energy minimization and thermal equilibration, with periodic boundary conditions applied as described in previous studies [76–79]. Subsequently, 3 independent replicates of 100-ns (resulting in a total simulation time of 3.6 μs) production MD simulations were performed for each M^pro^ complex using a 2-fs time step, with trajectory frames recorded every 5,000 steps. Short-range nonbonded interactions were managed using a 12-Å cutoff and a 10-Å switching distance. For long-range electrostatic interactions, the Particle mesh Ewald (PME) method was applied with a grid spacing of 1 Å, as described in previous studies [80–82]. MD simulation trajectories were analyzed using the available tools in Visual Molecular Dynamics (VMD) [83]. Full, aligned trajectories were used for all the MD simulation analyses. RMSD and RMSF analyses were performed on the basis of the Cα atoms of each residue to assess structural stability and flexibility. The binding free energy was estimated using the molecular mechanics Poisson–Boltzmann surface area method [62] implemented via the CaFE 1.0 plugin [84] integrated with VMD. Free energy change of binding (Δ*G*) values were determined for each 10-ns-long segments of the 100-ns-long trajectory, using frames extracted at 2-ns intervals (with a stride of 2 ns). H-bond analysis was carried out using the “Hydrogen Bonds” plugin in VMD, with a cutoff distance of 3.5 Å and a donor–hydrogen–acceptor (D–H···A) angle threshold of 20°. Interdomain H-bond occupancy differences between apo and holo M^pro^ complexes were evaluated using a 2-tailed Student’s *t* test. Each M^pro^–substrate peptide complex was compared individually against the apo M^pro^ dimer. DCC analyses, based on the positional fluctuations of Cα atoms, were performed using the MD-TASK software suite [85].

### Machine learning and SHAP analysis of substrate-catalytic interactions

To quantitatively assess the relationship between substrate site dynamics and catalytic site residue flexibility, we used a supervised machine learning framework integrating Random Forest Regression [65] with SHAP for model interpretability [66]. The dataset consisted of time-averaged RMSF values per residue obtained from MD simulations. For each residue, RMSF values were computed as the time average over the trajectory for each monomer. Each dimer has 2 monomers, and each system was simulated in 3 independent replicates, resulting in a total of 6 RMSF values per residue, which were treated as separate data points in the analysis. RMSF values of substrate peptide residues were used as predictive input features (X features), while those of catalytic site residues served as target outputs (Y features). Data preprocessing and analysis were conducted using Pandas and NumPy libraries in Python. For each catalytic site residue, an independent Random Forest Regressor (n_estimators = 100, random_state = 0) was trained to model the relationship between substrate peptide residue fluctuations and catalytic site residue flexibility. Model performance was assessed using the coefficient of determination (*R*^2^), which measures the proportion of variance in the observed values explained by the model. To interpret the relative contribution of individual substrate peptide residue, we applied SHAP, which assigns additive values (SHAP values) to each input feature, thereby quantifying its influence on the prediction of the model for each catalytic site residue (target). Importantly, the models were not used for external prediction but rather as a tool to reveal patterns and interactions within the dataset that may not be apparent from linear analysis.

### Data analysis and figure preparation

GraphPad Prism (GraphPad Software, La Jolla, CA, USA; www.graphpad.com), in combination with Microsoft Excel, was used for data analysis and graph preparation. Inkscape (Inkscape-1.1 version open-source software licensed under the GPL) was used to label and assemble the images.

## Conclusion

To conclude, MD simulations combined with supervised machine learning revealed differences in the localized structural dynamics of SARS-CoV-2 M^pro^ when it is bound to different substrate peptides. Specifically, substrate peptide binding altered the flexibility of the 2 regions (residues T45, S46, M49, and R188 to Q192), as reflected in RMSF profiles and SHAP contributions. Importantly, H-bond analysis showed not only variation in catalytic pocket interactions but also clear differences in interdomain H-bond interactions, Q189–M49, N28–C145, N28 (side ND2)–C145, Y54–D187, Q19–N119, Q83–G178, and N84–G178. The positioning of the catalytic pocket at the interface of these domains suggests that substrate binding may regulate protease activity by modulating these interdomain interactions. These insights provide a structural basis for understanding substrate-dependent dynamics and highlight interdomain H-bond interactions affected by substrate peptide binding. These structural insights can be used to further understand the cleavage of distinct M^pro^ substrate peptides and can be potentially valuable in antiviral drug design. Furthermore, the approach used for the analysis of MD simulations and the integration of machine learning (SHAP analysis on RMSF data) for understanding M^pro^–substrate peptide interactions can be readily extended to studies of other proteins.

## Data Availability

All data supporting the findings reported in this study are available within the article or the Supplementary Materials.
