# Supplementary material for: Differential Impact of Substrate Peptides on Interdomain Interactions in Severe Acute Respiratory Syndrome Coronavirus 2 Main Protease
Source: Comput Struct Biotechnol J. 2026 Apr 21;35(1):0058. doi: 10.34133/csbj.0058 (PMC13183263; doi:10.34133/csbj.0058)

Supproting Figure 1

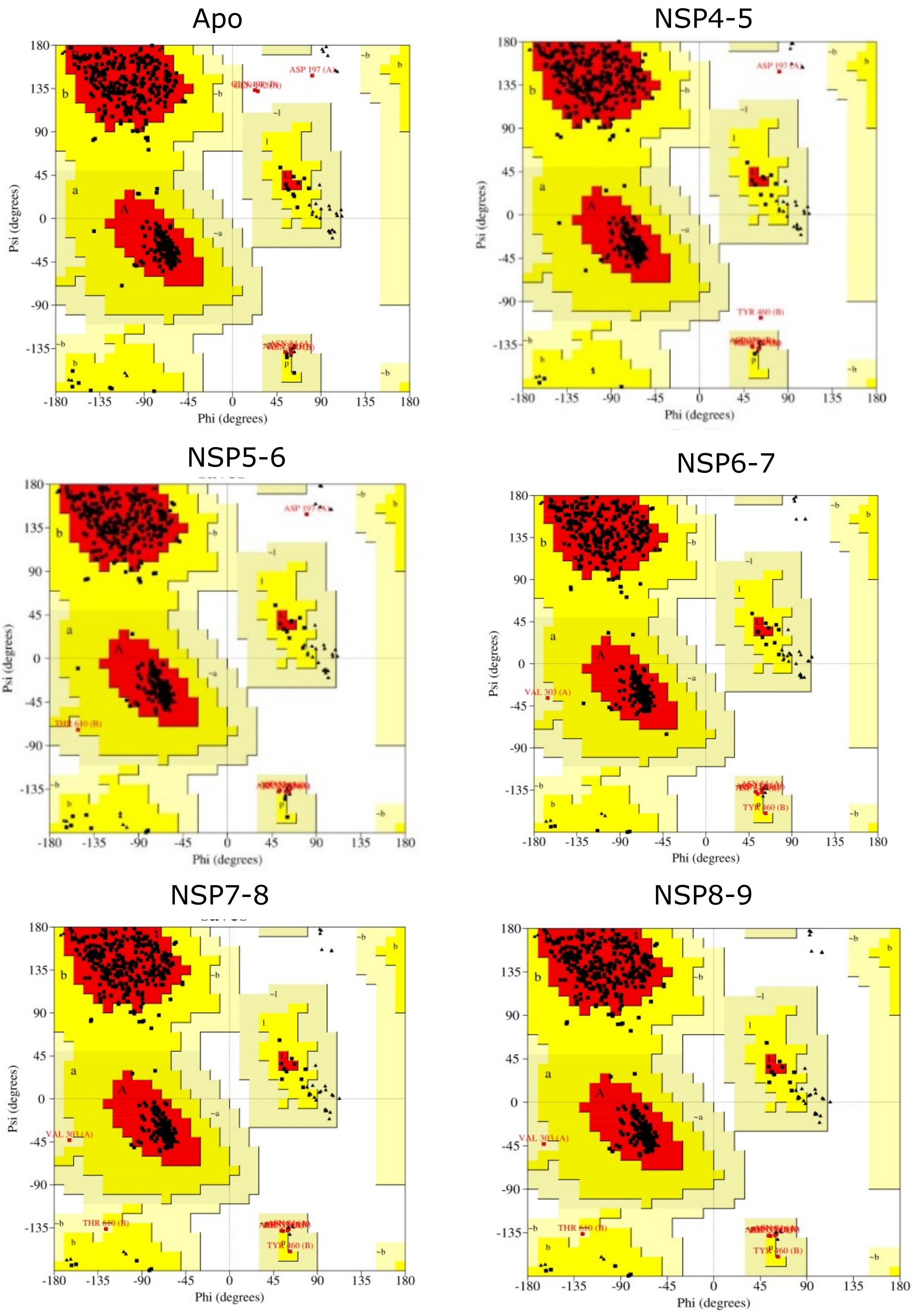

Supproting Figure 2

NSP9-10

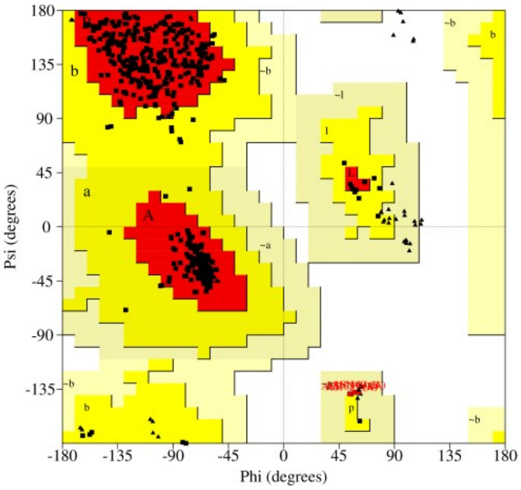

NSP10-11-12

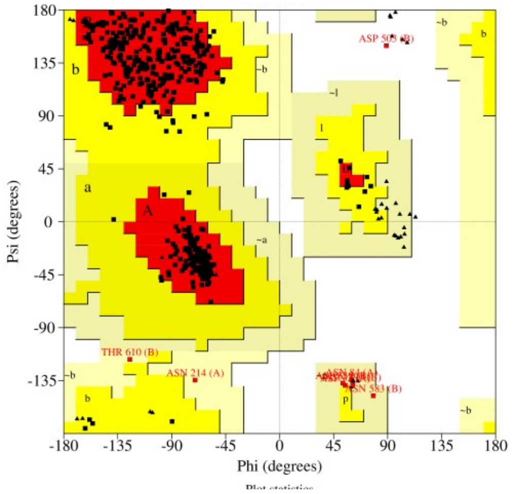

NSP12-13

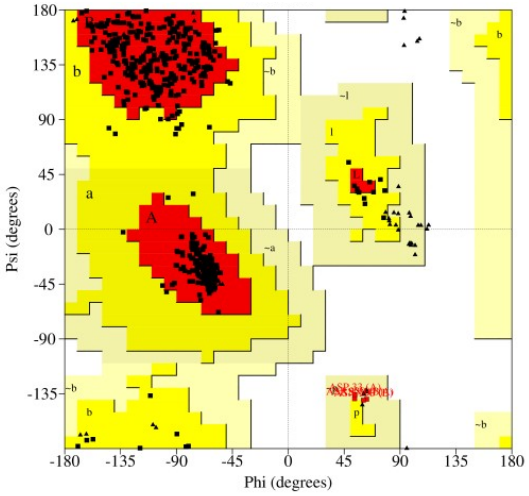

NSP13-14

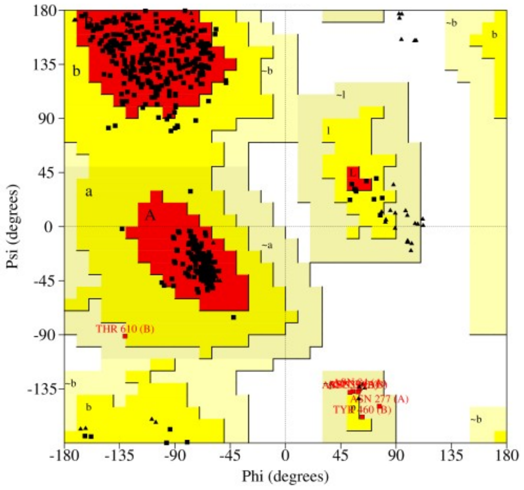

NSP14-15

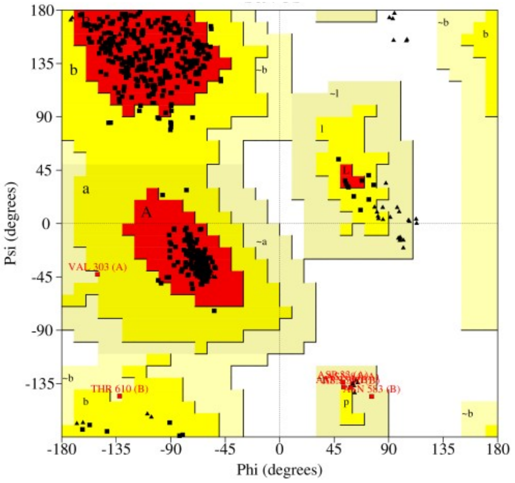

NSP15-16

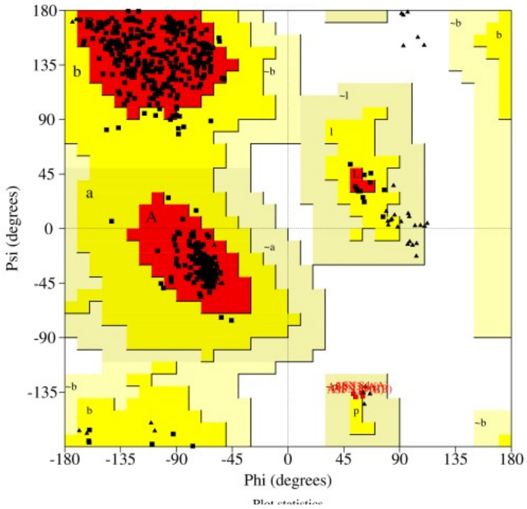

Supproting Figure 3

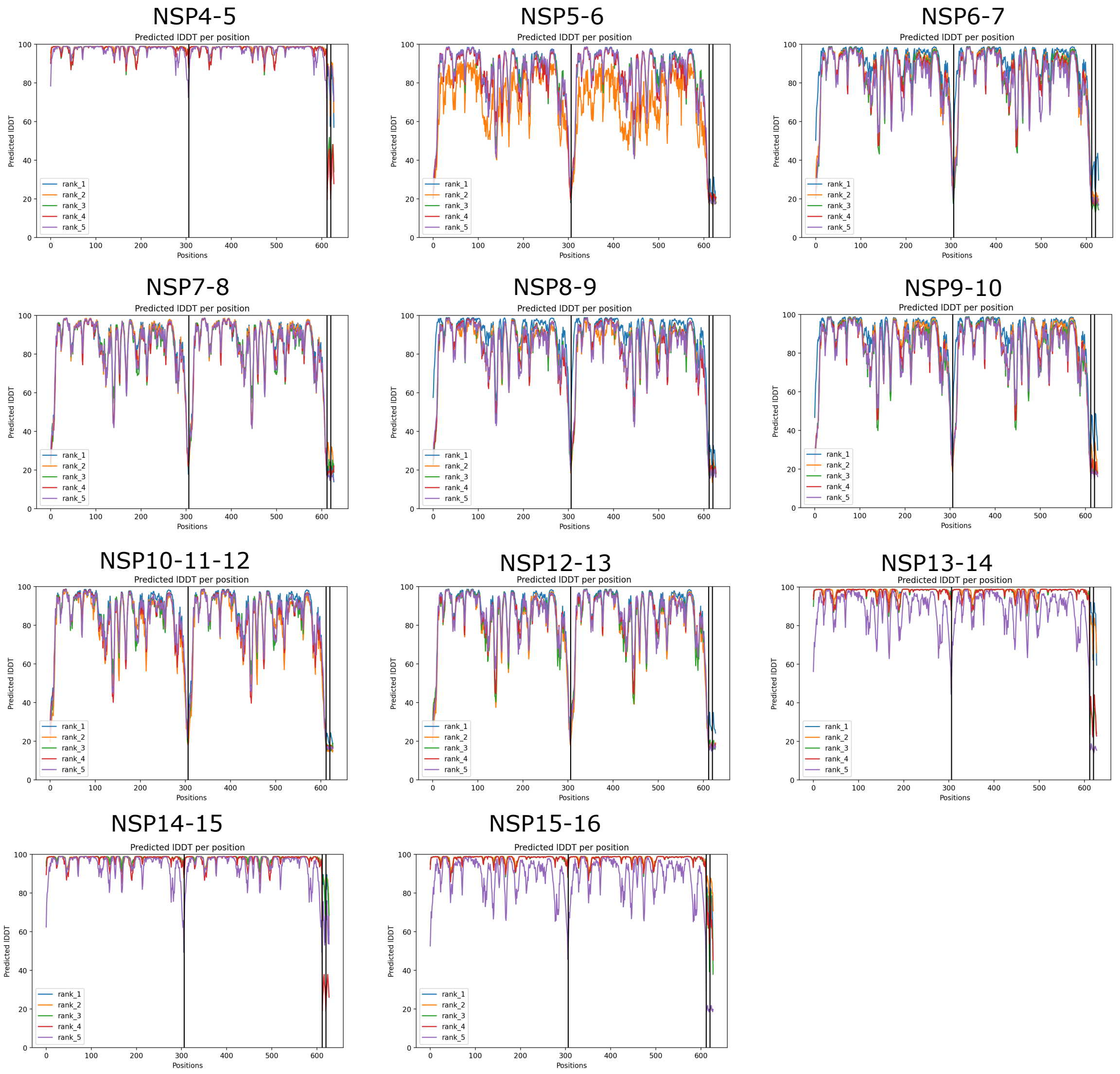

Supproting Figure 4

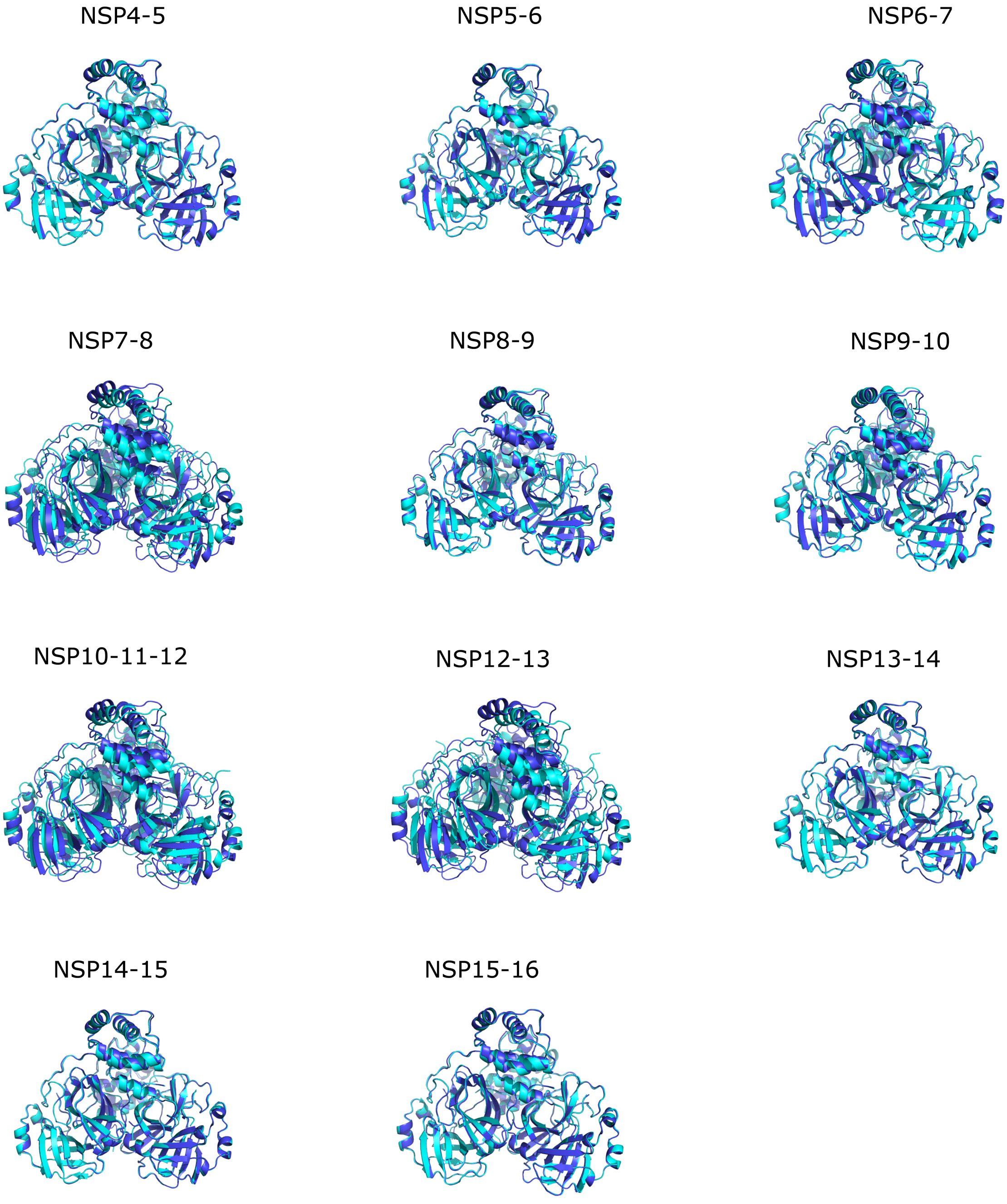

Supproting Figure 5

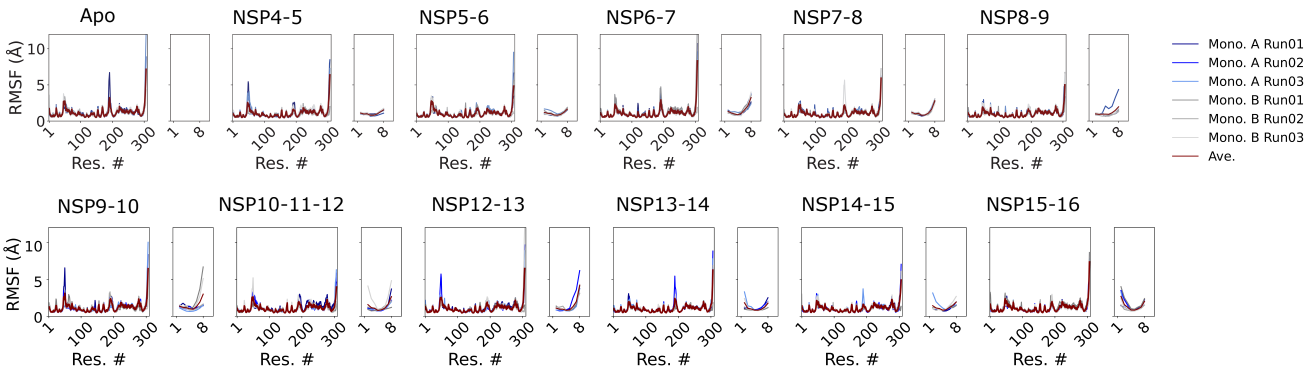

Supproting Figure 6

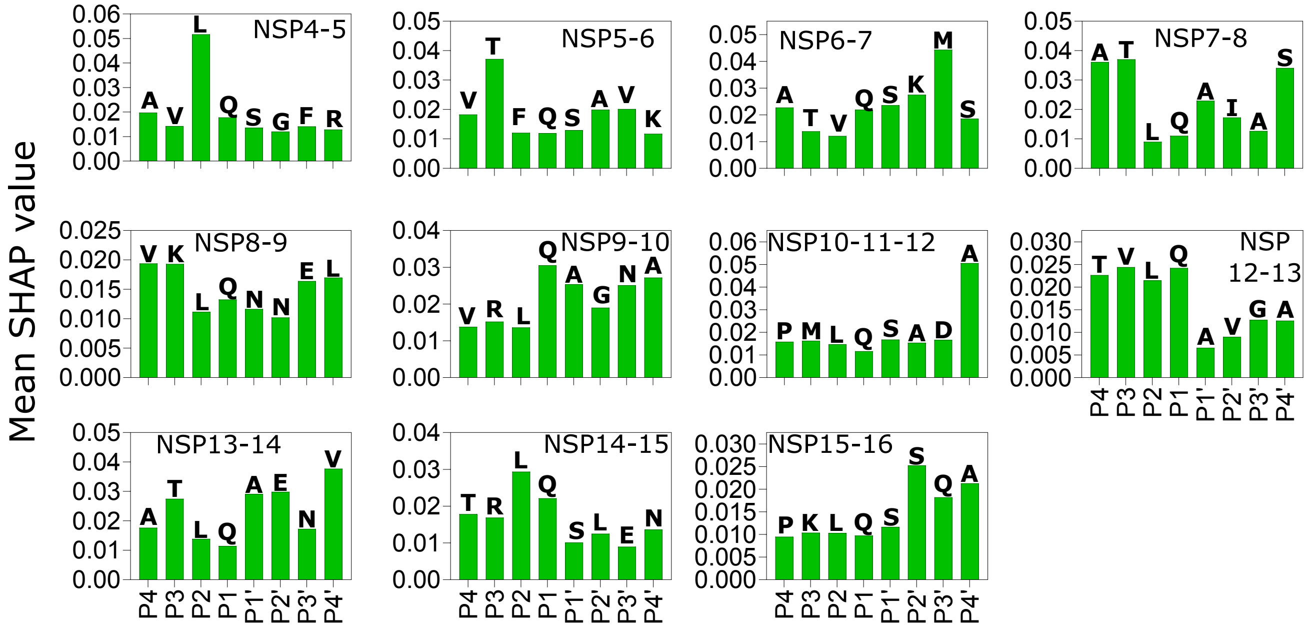

Supproting Figure 7

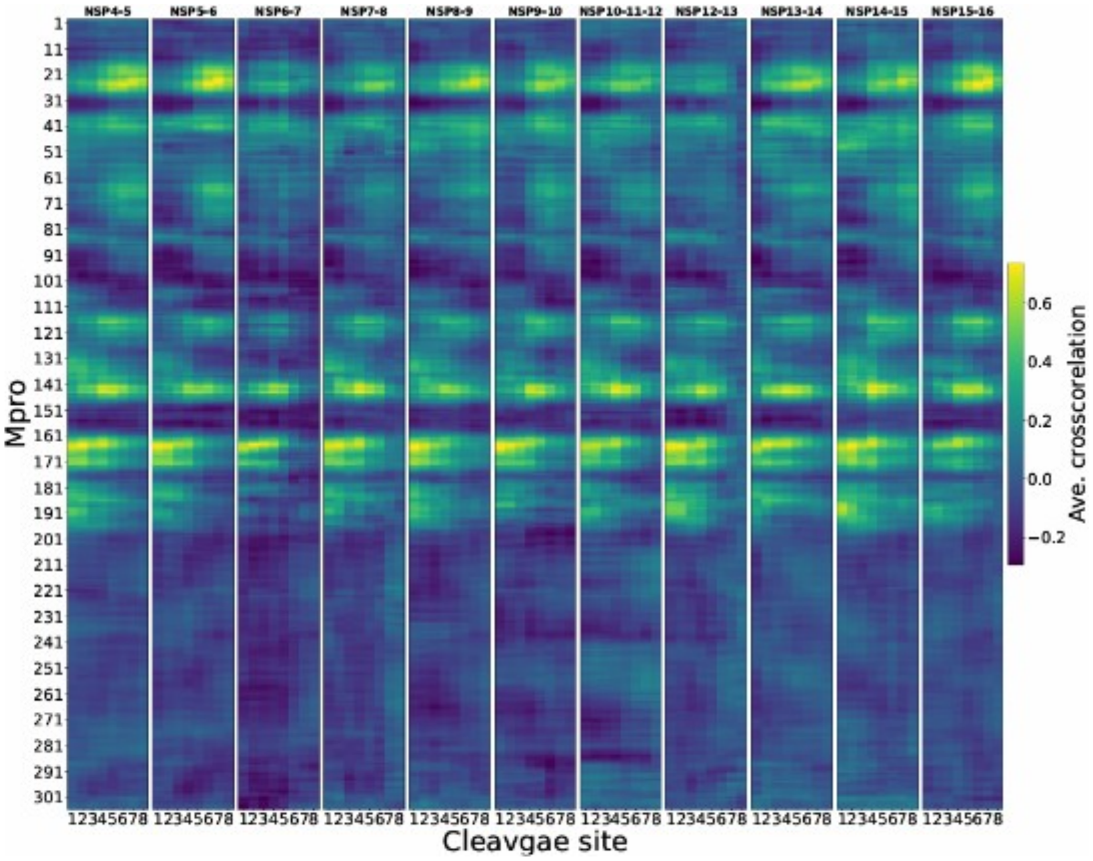

Supproting Figure 8

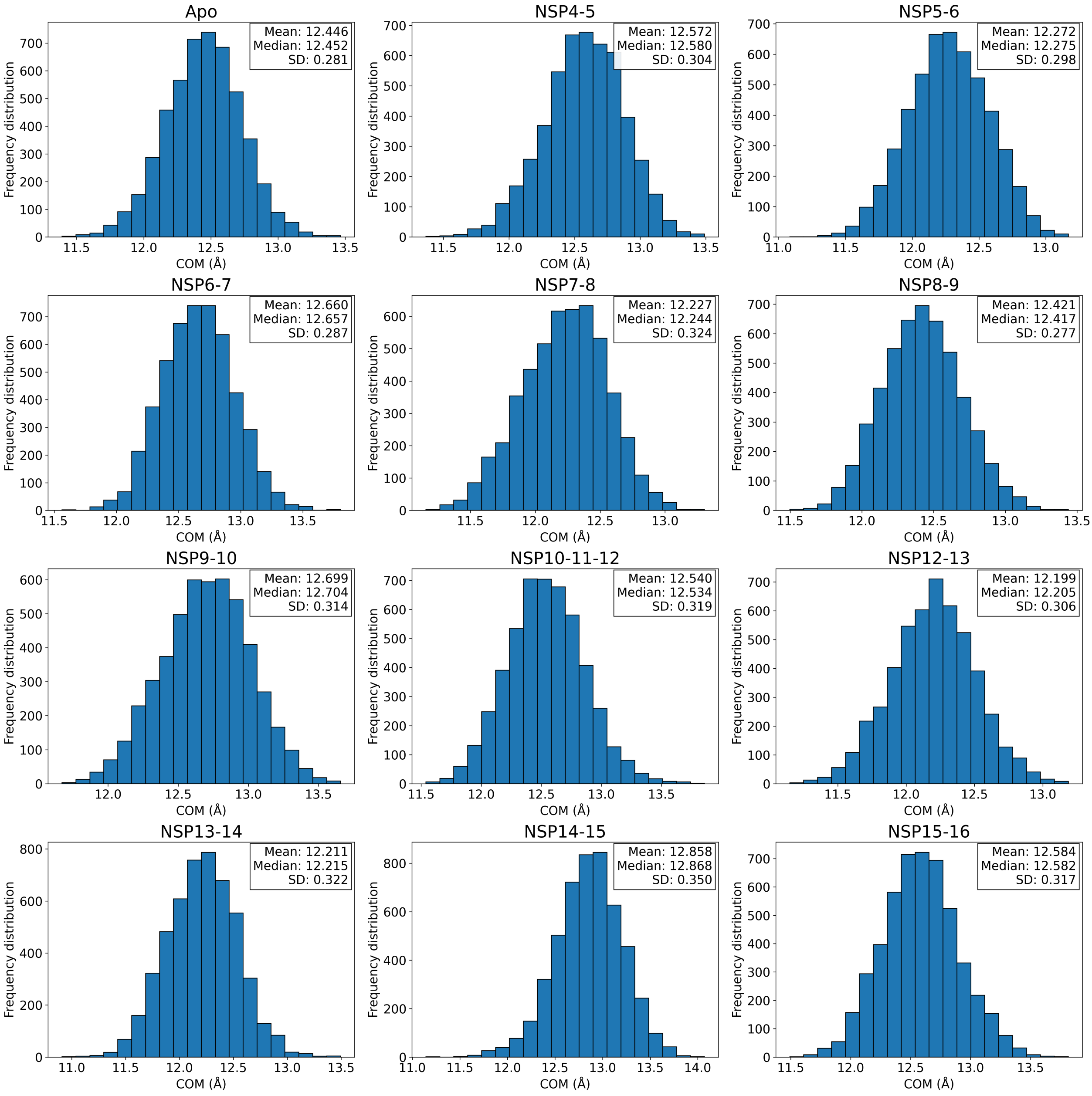

Supproting Figure 9

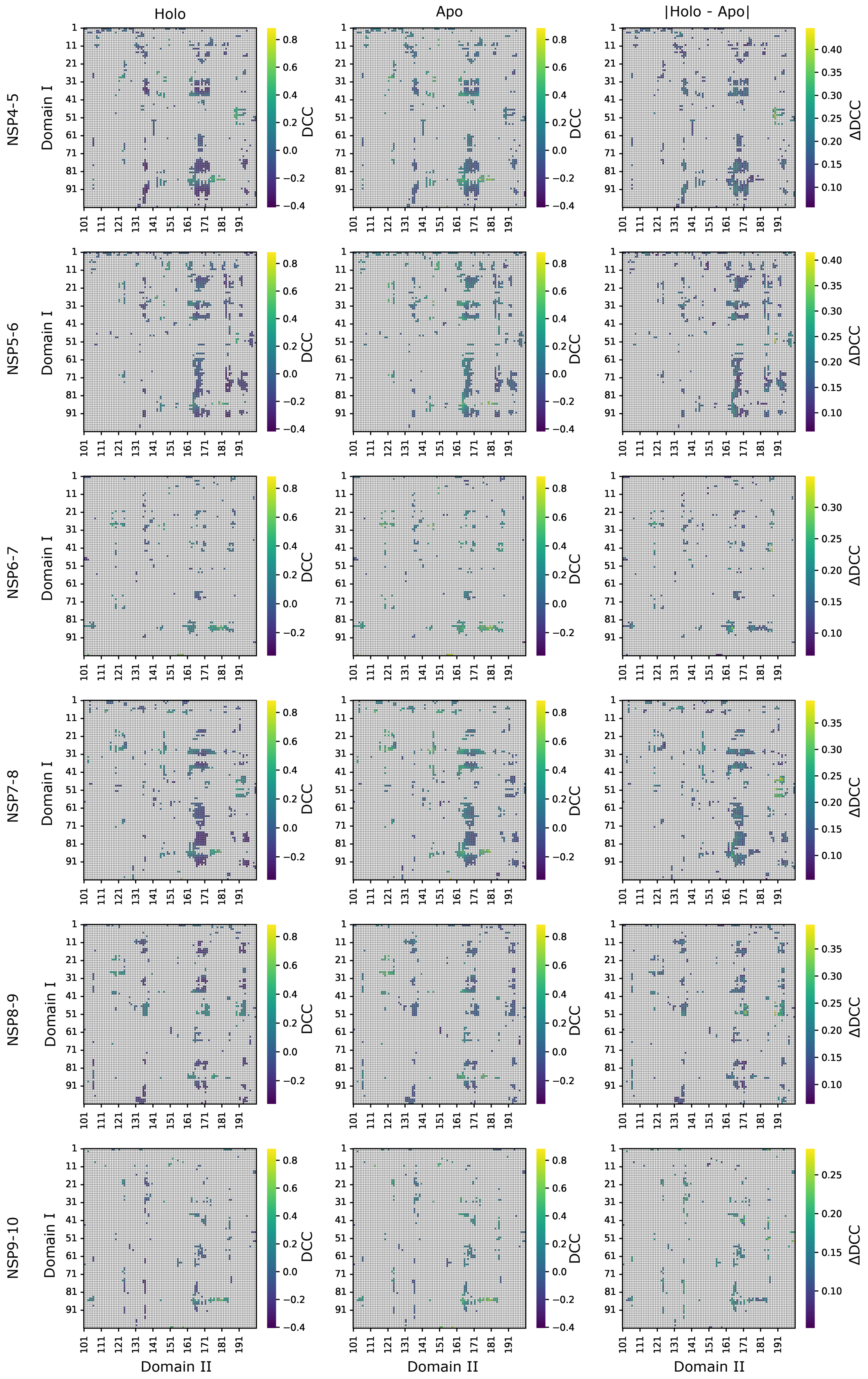

Supproting Figure 10

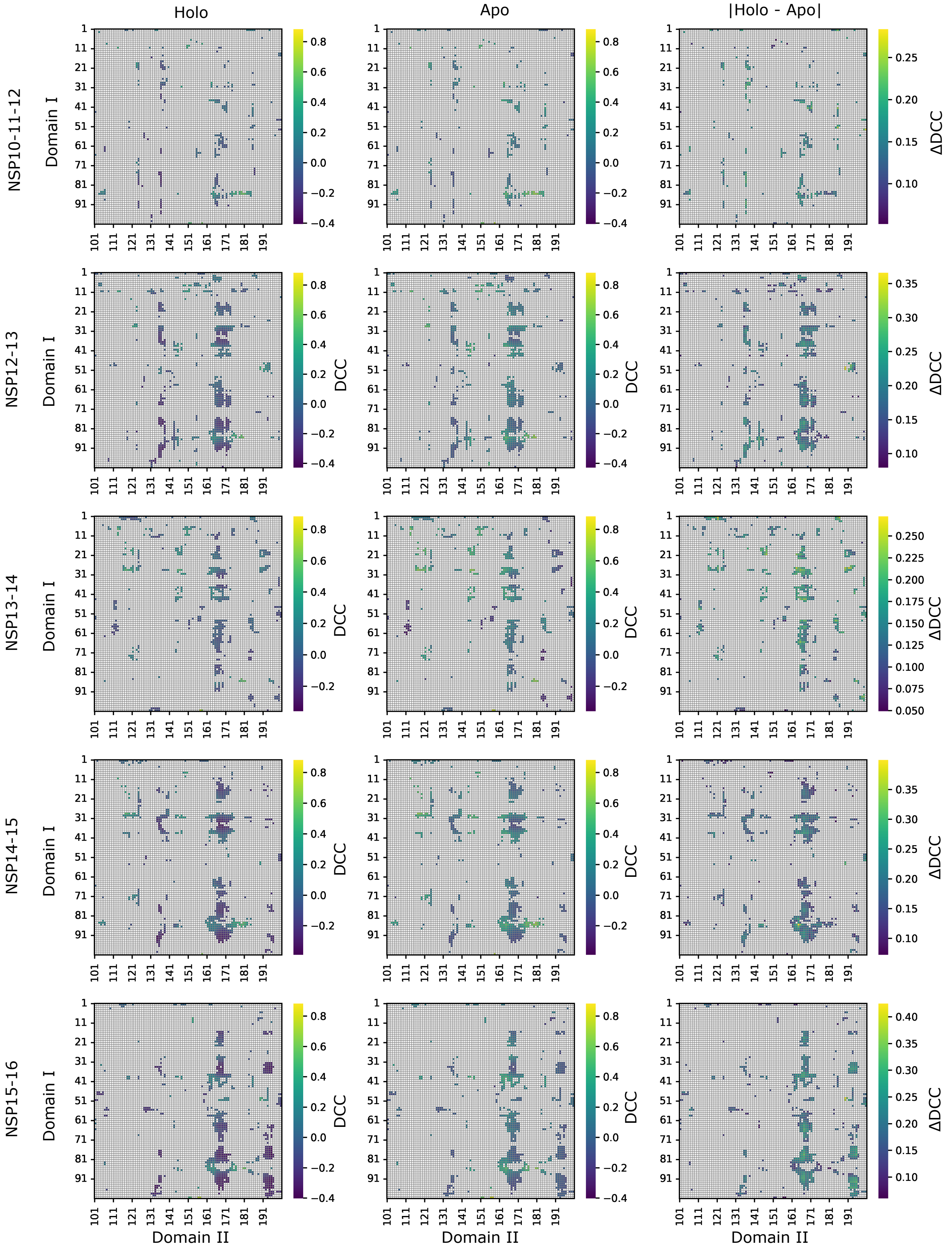

Supproting Figure 11

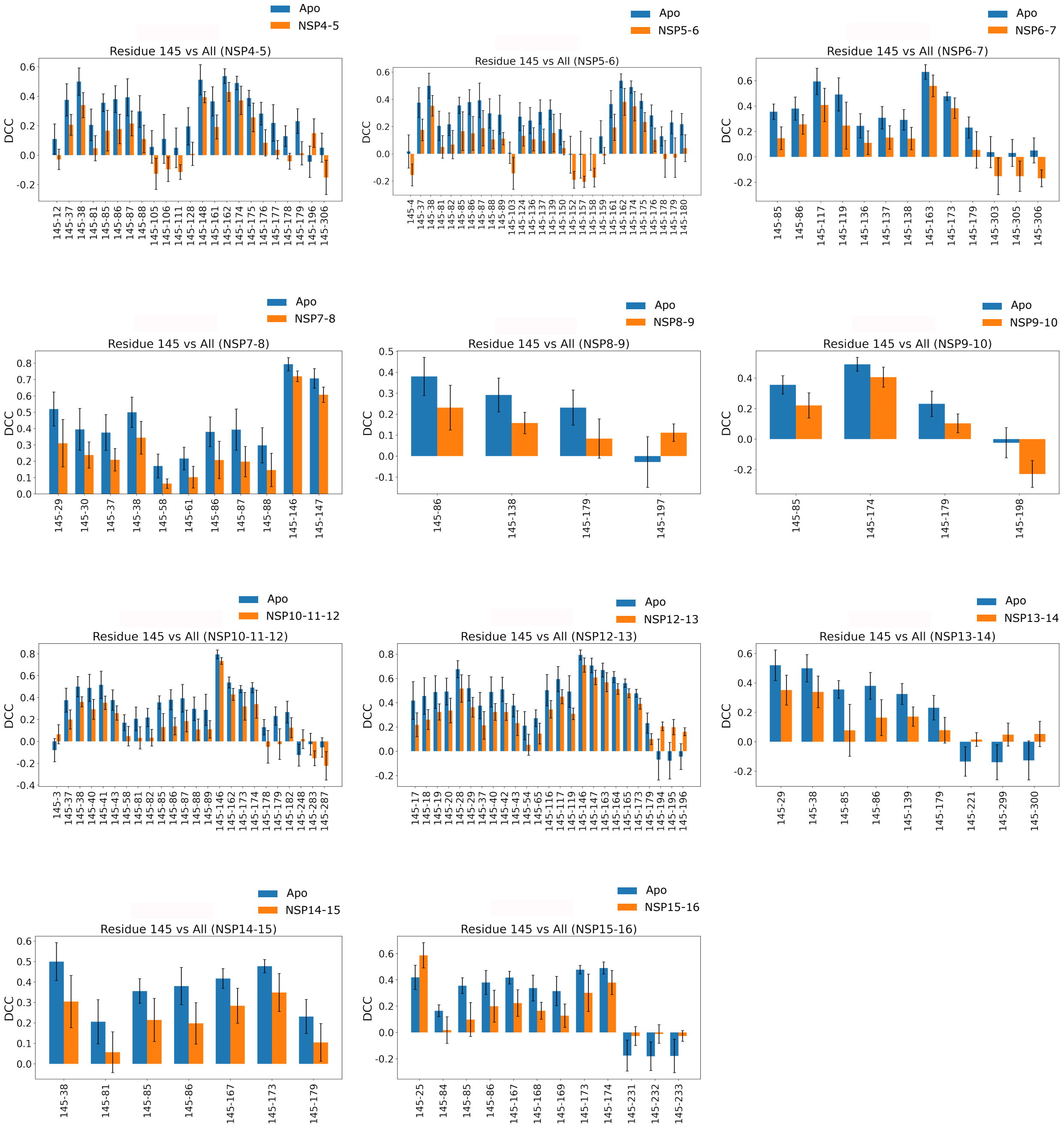

Supplement: Supplementary 1 — Tables S1 to S5 Figs. S1 to S11 [file csbj.0058.f1.zip › SI-figures.pdf]
