# Supplementary material for: Differential Impact of Substrate Peptides on Interdomain Interactions in Severe Acute Respiratory Syndrome Coronavirus 2 Main Protease
Source: Comput Struct Biotechnol J. 2026 Apr 21;35(1):0058. doi: 10.34133/csbj.0058 (PMC13183263; doi:10.34133/csbj.0058)
Supplement: Supplementary 1 — Tables S1 to S5 Figs. S1 to S11 [file csbj.0058.f1.zip › Supporting Information.docx]

# **Supporting Information**

**Differential Impact of Substrate Peptides on Interdomain Interactions in SARS-CoV-2 M^pro^**

Asma Fatima^1^, Kabir H Biswas^1,*^

**Affiliation**:

^1^College of Health & Life Sciences, Hamad Bin Khalifa University, Doha, Qatar

ORCID:

Kabir H Biswas: 0000-0001-9194-4127

Asma Fatima: 0009-0004-7191-5109

***Correspondence:** [kbiswas@hbku.edu.qa](mailto:kbiswas@hbku.edu.qa)

**Keywords**: SARS-CoV-2; M^pro^; Interdomain Interactions; MD simulation; Machine Learning;

## Supporting Tables

**Supporting Table 1.** Table showing DOPE scores [54] of modeled protein structures generated by Modeller. The lowest DOPE scores, highlighted in bold, represent the best-scoring models for the respective M^pro^ structural model, which were selected and utilized for further analysis.

**Supporting Table 2.** Table showing the pLDDT values AlphaFold2-derived M^pro^-substrate peptide complexes. Sub. Pep., substrate peptide.

**Supporting Table 3.** Table showing RMSD values between Modeller- and AlphaFold2-derived M^pro^–substrate peptide complex structural models.

**Supporting Table 4.** Table showing the R² values obtained from the machine learning model performance evaluation.

**Supporting Table 5.** Table showing H-bond occupancy from the interdomain H-Bond analysis. *p*-values were obtained from Student’s t-test by comparing the indicated M^pro^-substrate peptide complex against the apo M^pro^ trajectory.

## Supporting Figures

**Supporting Figure 1. Ramachandran plots of the structural models of apo dimeric M^pro^ and in complex with NSP4-5, NSP5-6, NSP6-7, NSP7-8 and NSP8-9 substrate peptides.**

Ramachandran plots for the modeled apo and substrate peptide-bound M^pro^ structures, generated using PROCHECK, show the distribution of backbone dihedral angles (φ and ψ) for all residues in the structural models. Residues in the most favored regions are shown in red. Allowed regions are shown in yellow, generously allowed regions in light yellow, and disallowed regions in white. All structural models show residues predominantly within favored regions, confirming their good stereochemical quality.


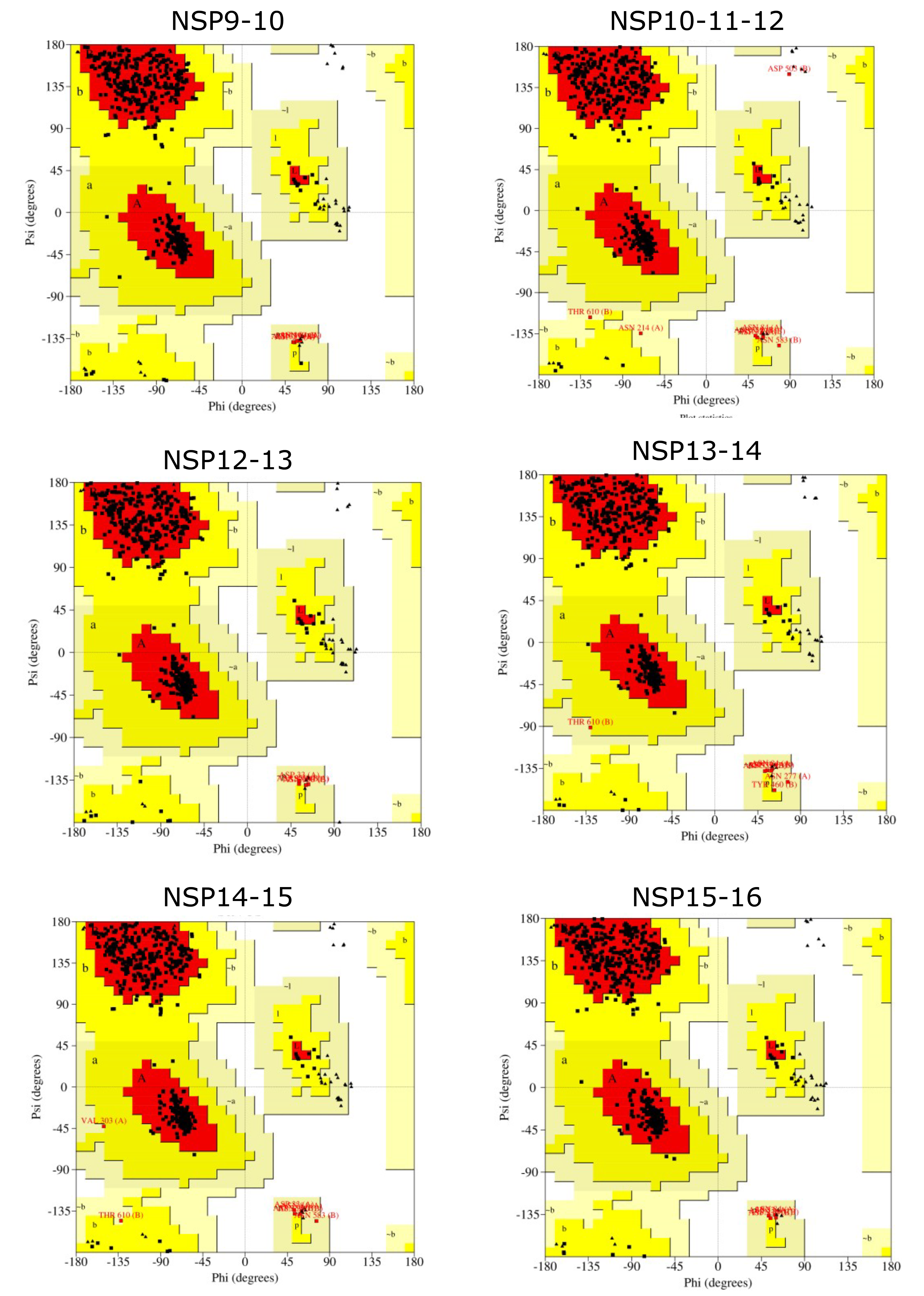


**Supporting Figure 2. Ramachandran plots of the structural models of dimeric M^pro^ with NSP9-10, NSP10-11-12, NSP12-13, NSP13-14, NSP14-15 and NSP15-16 substrate peptides.**

Ramachandran plots for the modeled apo and substrate peptide-bound M^pro^ structures, generated using PROCHECK, show the distribution of backbone dihedral angles (φ and ψ) for all residues in the structural models. Residues in the most favored regions are shown in red. Allowed regions are shown in yellow, generously allowed regions in light yellow, and disallowed regions in white. All structural models show residues predominantly within favored regions, confirming their good stereochemical quality.


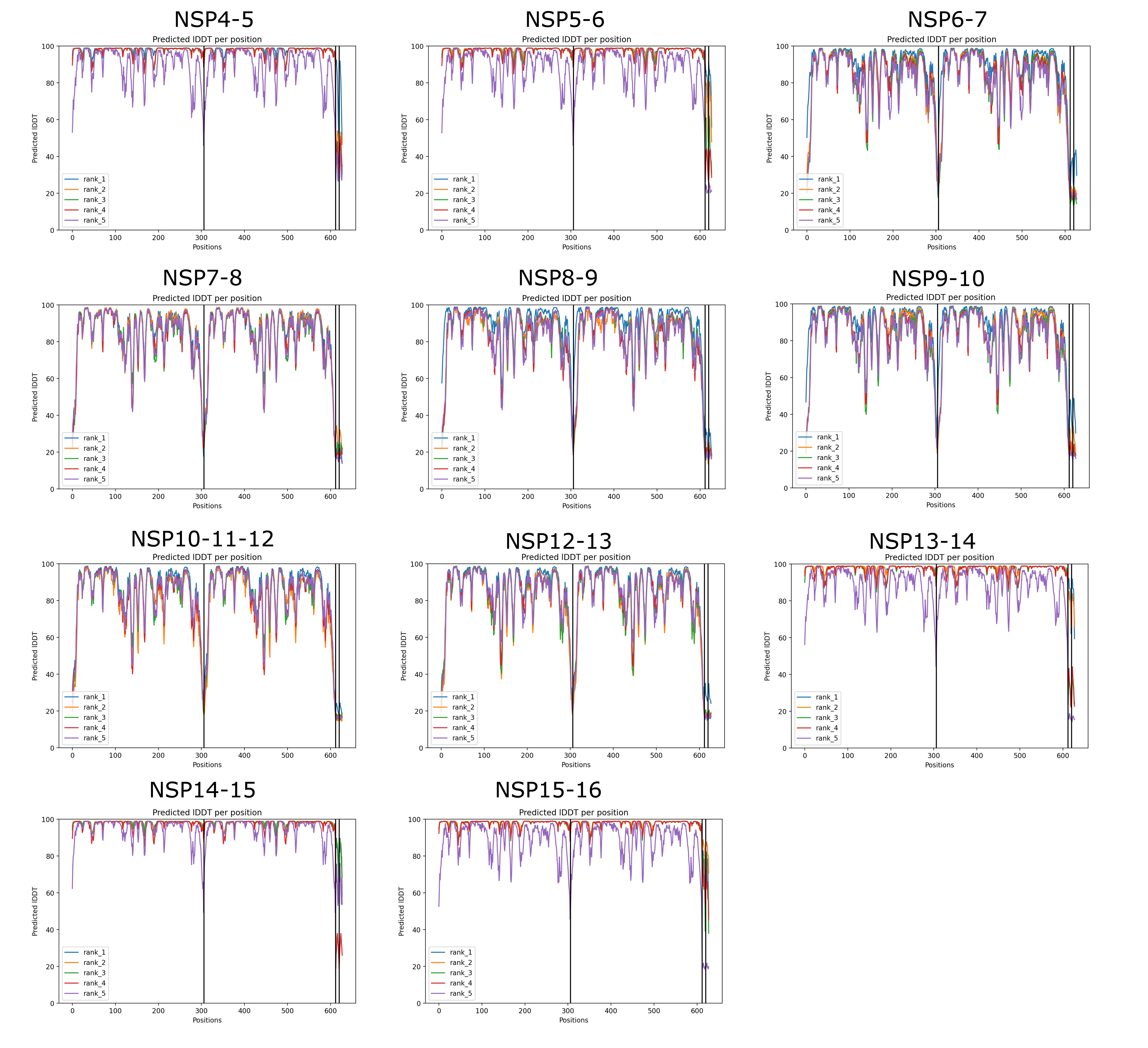


**Supporting Figure 3. Confidence scores for predicted M^pro^-substrate peptide complexes using AlphaFold2.**

Per-residue predicted Local Distance Difference Test (pLDDT) scores are shown for M^pro^ dimers in complex with eleven distinct substrate peptides (NSP4-5 to NSP15-16). Residues 1-306 and 307-612 represents monomer 1 and 2, respectively, of the M^pro^ dimer, while residues 612-620 and 621-628 represent the substrate peptides bound to monomer 1 and 2, respectively.


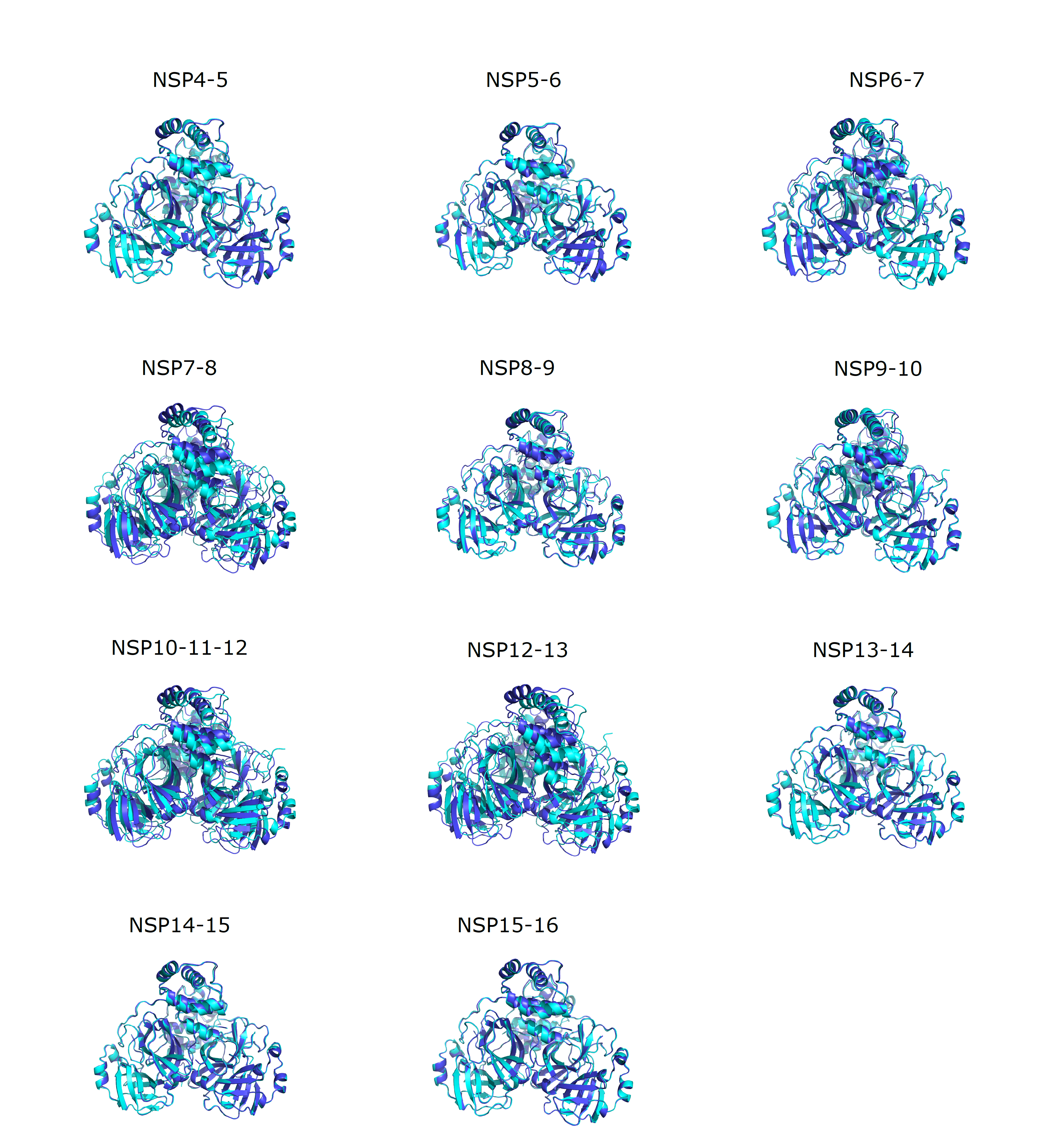


**Supporting Figure 4. Structural alignment of M^pro^-substrate peptide complexes was generated with Modeller and AlphaFold2.**

Comparison of M^pro^–substrate peptide models generated via AlphaFold2 (cyan) and Modeller (dark blue). For each of the eleven substrate peptides (NSP4-5 to NSP15-16), the models were aligned in PyMOL to assess structural similarity.

**Supporting Figure 5. RMSF analysis of M^pro^-substrate peptide complexes.**
Graphs showing RMSF values of individual monomers (left panels) and substrate peptides, if present, (right panel) from the apo- or indicated dimeric M^pro^–substrate peptide complexes obtained from three independent, 100 ns long MD simulations.


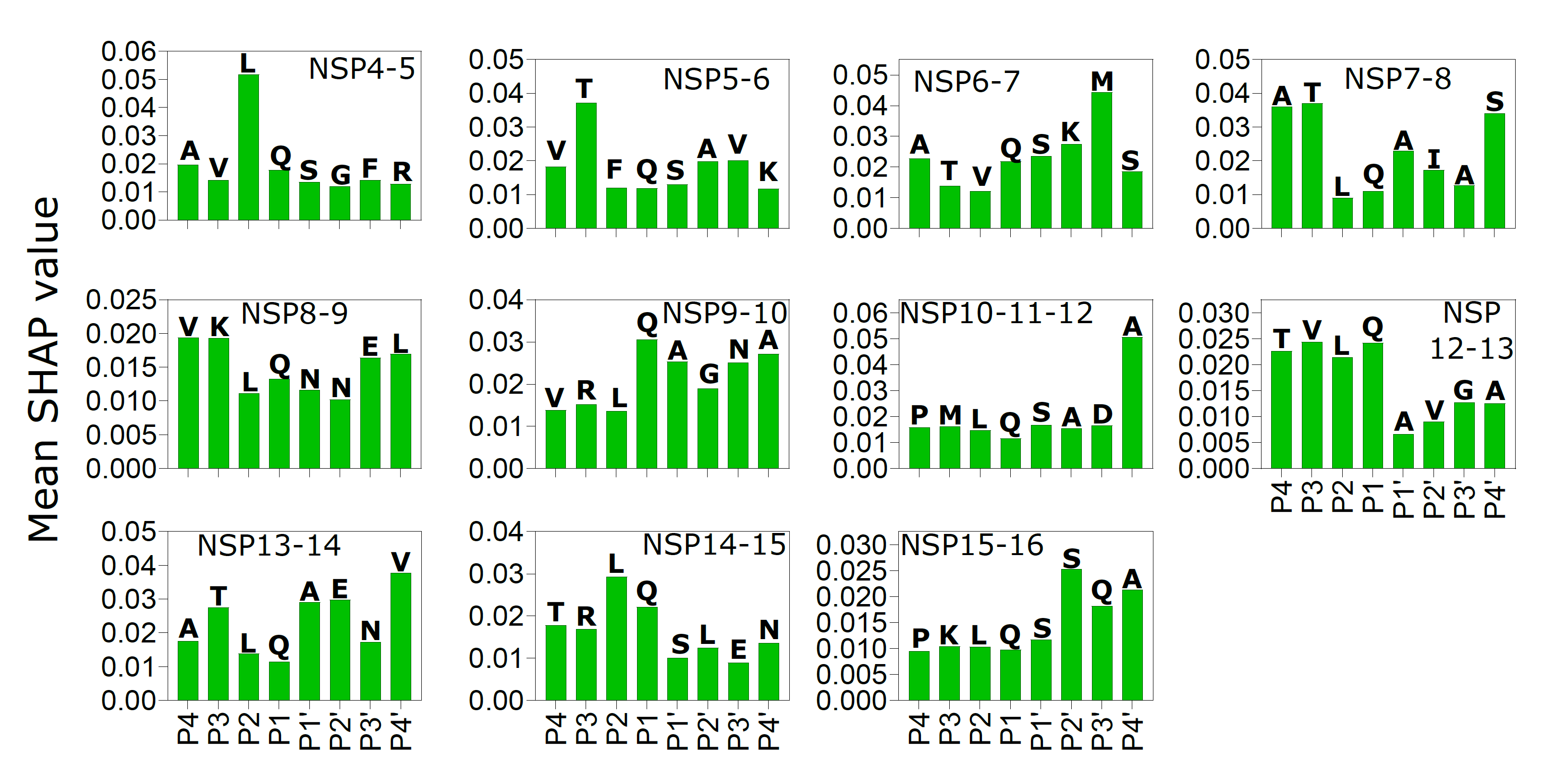


**Supporting Figure 6. Mean SHAP values of M^pro^-substrate peptide residues.**

Graphs showing mean SHAP values of substrate peptide residues derived from the machine learning model trained on the RMSF values of M^pro^ catalytic site and substrate peptide residues.

**
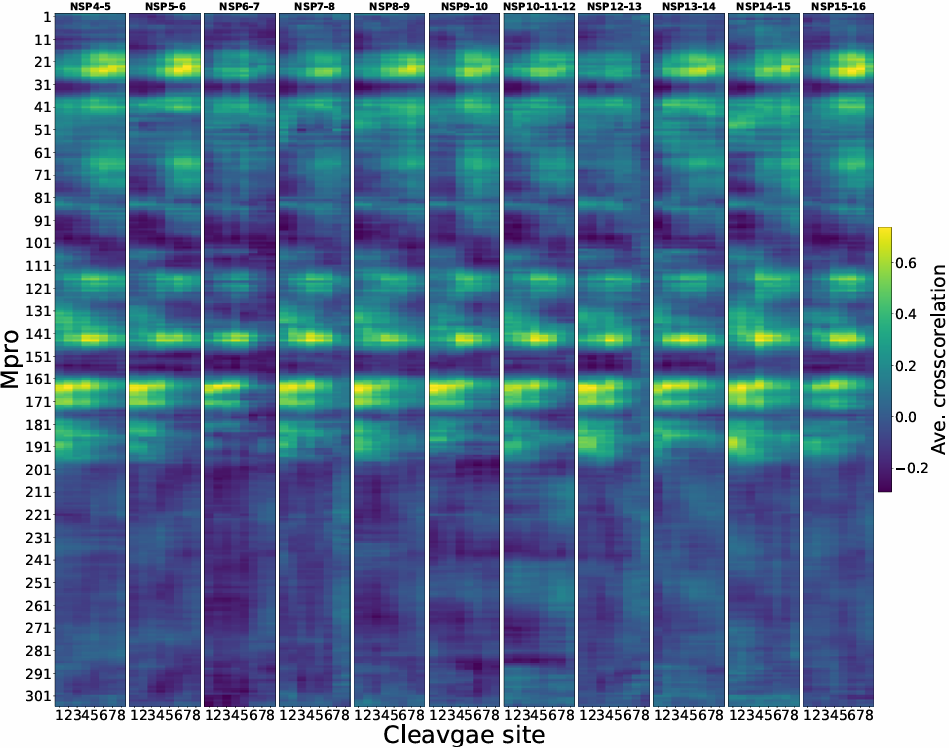
**

**Supporting Figure 7. Dynamic cross-correlation (DCC) values between M^pro^ and substrate peptide residues.**Heatmaps showing DCC values calculated between the M^pro^ and the substrate peptide residues. Values shown were obtained from averaging DCC values of the two monomers and from three independent, 100 ns long MD simulations.

**
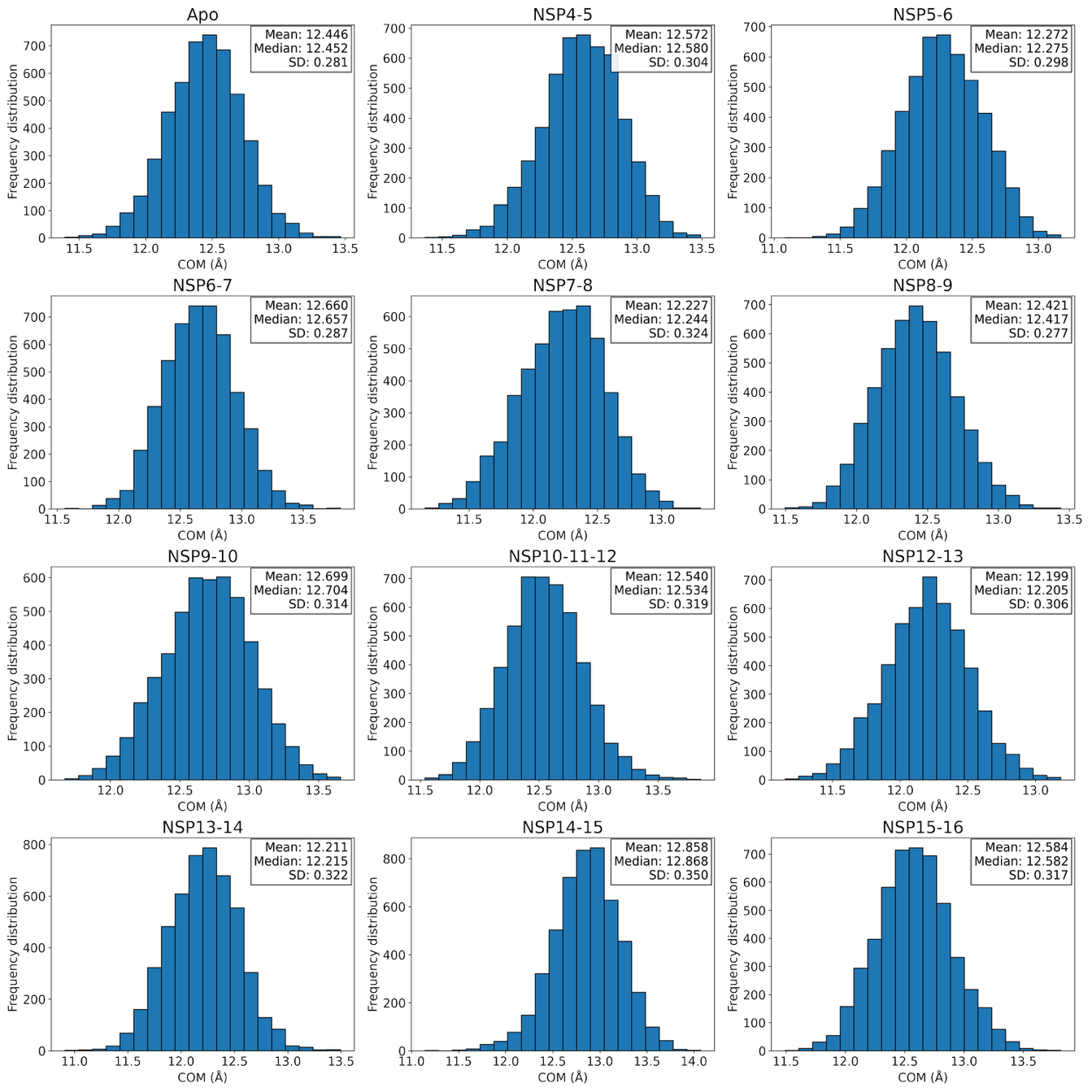
**

**Supporting Figure 8. Interdomain center of mass (COM) distance distribution**

Graph showing frequency distributions of the COM distances between domains I and II for apo M^pro^ and M^pro^ in complex with the indicated substrate peptides. The frequency distributions shown were obtained from average of the COM distance from each monomer and three independent, 100 ns long MD simulations.

**Supporting Figure 9. Substrate-dependent modulation of interdomain correlated motions in M^pro^ in complex with NSP4-5, NSP5-6, NSP6-7, NSP7-8, NSP8-9 and NSP9-10 substrate peptides.**

Heat maps showing changes in residue-residue correlated motions between domains I and II of M^pro^ in complex with the indicated substrate peptides. The left panel shows the average DCC values of residue pairs that exhibit statistically significant differences relative to the apo M^pro^. The middle panel shows the corresponding DCC values for the same domain I–domain II residue pairs in the apo M^pro^. The right panel displays the absolute difference in DCC values between apo and holo M^pro^ states for the corresponding residue pairs.

Data shown are the average DCC obtained from the three independent, all-atom 100 ns MD simulations. Statistical significance was determined using students t-test, with p < 0.05 considered significant, and only DCC of residue pairs meeting this threshold are shown.

**Supporting Figure 10. Substrate-dependent modulation of interdomain correlated motions in M^pro^ in complex with NSP10-11-12, NSP12-13, NSP13-14, NSP14-15 and NSP15-16 substrate peptides.**

Heat maps showing changes in residue-residue correlated motions between domains I and II of M^pro^ in complex with the indicated substrate peptides. The left panel shows the average DCC values of residue pairs that exhibit statistically significant differences relative to the apo M^pro^. The middle panel shows the corresponding DCC values for the same domain I–domain II residue pairs in the apo M^pro^. The right panel displays the absolute difference in DCC values between apo and holo M^pro^ states for the corresponding residue pairs.

Data shown are the average DCC obtained from the three independent, all-atom 100 ns MD simulations. Statistical significance was determined using students t-test, with p < 0.05 considered significant, and only DCC of residue pairs meeting this threshold are shown.


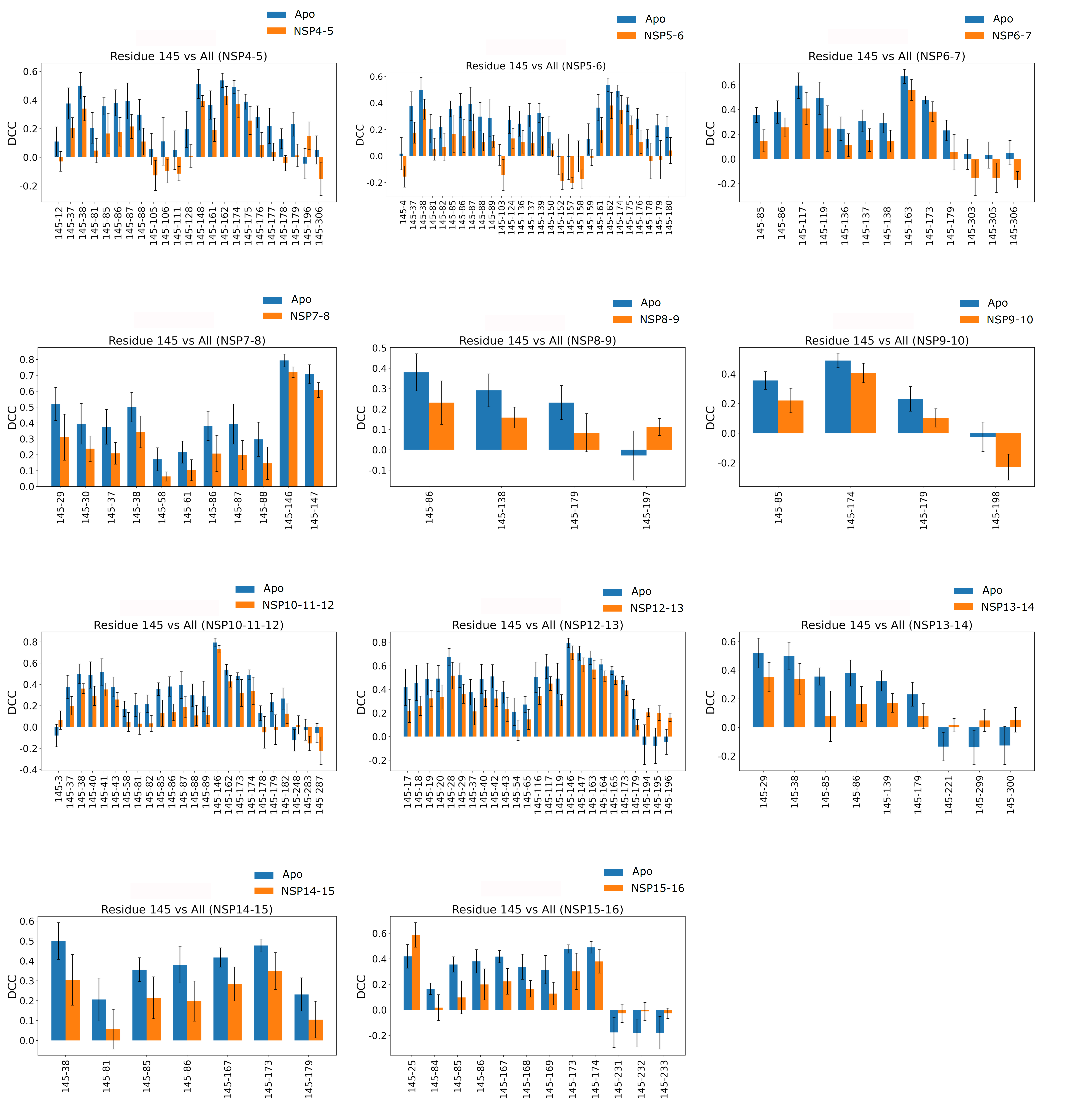


**Supporting Figure 11. Residues exhibiting significant changes in dynamic correlations with the catalytic residue C145 of M^pro^.**Graphs showing DCC values of residues that showed statistically significant differences in DCC of Cα atomic motions with the catalytic residue C145 upon substrate binding between apo M^pro^ dimer and M^pro^ dimer in complex with the indicated substrate peptide. Residues were selected based on a significance threshold of p < 0.05, determined using Student’s t-test.
